# Differential condensation of sister chromatids acts with Cdc6 to ensure asynchronous S-phase entry in *Drosophila* male germline stem cell lineage

**DOI:** 10.1016/j.devcel.2022.04.007

**Published:** 2022-04-27

**Authors:** Rajesh Ranjan, Jonathan Snedeker, Matthew Wooten, Carolina Chu, Sabrina Bracero, Taylar Mouton, Xin Chen

**Affiliations:** 1 Howard Hughes Medical Institute, Department of Biology, The Johns Hopkins University, Baltimore, MD 21218, USA; 2 Department of Biology, The Johns Hopkins University, Baltimore, MD 21218, USA; 3 Present address: Fred Hutchinson Cancer Research Center, Seattle, WA 98109-1024, USA; 4 Present address: University of Iowa, Carver College of Medicine, Iowa City, IA 52242, USA; 5 Present address: Department of Immunology, Harvard Medical School, Boston, MA 02115, USA; 6 These authors contributed equally; 7 Lead contact

## Abstract

During *Drosophila melanogaster* male germline stem cell (GSC) asymmetric division, preexisting old versus newly synthesized histones H3 and H4 are asymmetrically inherited. However, the biological outcomes of this phenomenon have remained unclear. Here, we tracked old and new histones throughout the GSC cell cycle through the use of high spatial and temporal resolution microscopy. We found unique features that differ between old and new histone-enriched sister chromatids, including differences in nucleosome density, chromosomal condensation, and H3 Ser10 phosphorylation. These distinct chromosomal features lead to their differential association with Cdc6, a pre-replication complex component, and subsequent asynchronous DNA replication initiation in the resulting daughter cells. Disruption of asymmetric histone inheritance abolishes differential Cdc6 association and asynchronous S-phase entry, demonstrating that histone asymmetry acts upstream of these critical cell-cycle progression events. Furthermore, disruption of these GSC-specific chromatin features leads to GSC defects, indicating a connection between histone inheritance, cell-cycle progression, and cell fate determination.

## In brief

*Drosophila* male germline stem cells (GSCs) undergo asymmetric cell division. Ranjan et al. find that GSC sister chromatids differ in nucleosome density, chromosomal condensation, and H3 Ser10 phosphorylation. These distinct features cause them to differentially recruit a key replication component, leading to asynchronous initiation of the subsequent cell cycle.

## Graphical Abstract

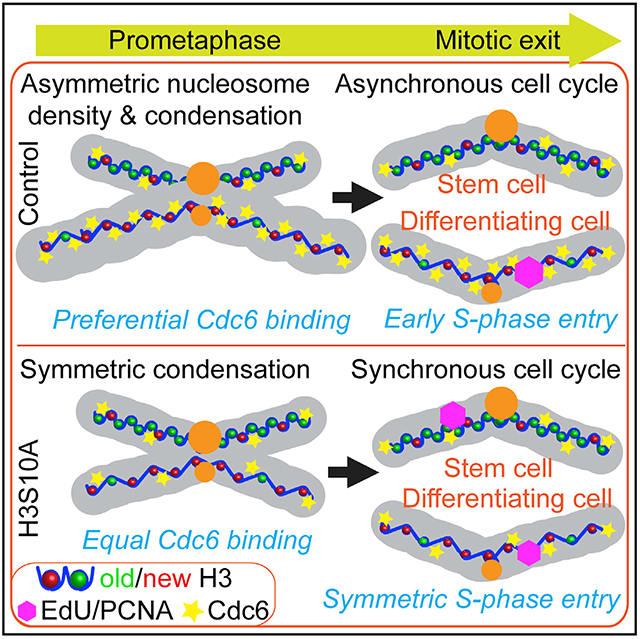

## INTRODUCTION

A fundamental question in developmental biology is how cells with identical genomes take on distinct cell fates. One important context for understanding cell fate decision is asymmetric cell division (ACD), wherein two daughter cells establish different cell fates following a single mitosis. ACD has been characterized in multiple organisms during development, homeostasis, and tissue regeneration (Sunchu and Cabernard, 2020; Venkei and Yamashita, 2018; Zion et al., 2020; Blanpain and Fuchs, 2014). Adult stem cells often undergo ACD to generate one self-renewing stem daughter cell and another differentiating daughter cell.

Epigenetic mechanisms play important roles in cell fate decisions by altering chromatin structure and gene expression patterns while preserving primary DNA sequences (Allis and Jenuwein, 2016). In eukaryotic organisms, chromatin is organized by nucleosome units of 145–147-bp DNA wrapped around a histone octamer composed of H3, H4, H2A, and H2B (Kornberg, 1974; Luger et al., 1997; Richmond and Davey, 2003). Histones are one major epigenetic information carriers, serving both to package DNA and to regulate gene expression through multiple mechanisms (Bannister and Kouzarides, 2011; Zhang et al., 2021; Stillman, 2018). The composition of the nucleosome array and the positioning of individual nucleosomes contribute to regulating both local gene expression and DNA replication timing (Struhl and Segal, 2013; Jiang and Pugh, 2009; Rhind and Gilbert, 2013; Allshire and Madhani, 2018; Kornberg and Lorch, 2020; Valouev et al., 2011). At regulatory DNA elements such as promotors, enhancers, and origins of replication, nucleosomes have been shown to directly compete with key regulators such as transcription factors and origin recognition complex (ORC) components for binding to DNA (Ramachandran and Henikoff, 2016; MacAlpine et al., 2010). For example, low nucleosome occupancy at enhancers and promotors allows for efficient transcription factor binding and gene activation, whereas high nucleosome occupancy at these regions often antagonizes binding of transcription factors, which results in gene silencing (West et al., 2014; Lai and Pugh, 2017). During cellular differentiation, changes in histone modifications, nucleosome densities, and nucleosome positioning alter chromatin structure in order to regulate gene expression, replication timing, and ultimately cell fate decisions (Belsky et al., 2015; Lipford and Bell, 2001; Rodriguez et al., 2017; Simpson, 1990; Goldberg et al., 2007; Meshorer and Misteli, 2006; Stancheva, 2011). Maintaining a unique chromatin structure could preserve stem cell identity while changing the chromatin landscape may lead to cellular differentiation, as has been shown in multiple stem cell lineages (Atlasi and Stunnenberg, 2017; Avgustinova and Benitah, 2016; Wooten et al., 2020b; Feng and Chen, 2015).

In stem cell lineages, cell fate changes and cell-cycle remodeling are often linked during stem cell differentiation, progenitor cell dedifferentiation, or trans-differentiation between cell types (Soufi and Dalton, 2016; Dalton, 2015; Hu et al., 2019; Guo et al., 2014). In particular, the length of G1 phase has been shown to be tightly associated with cell fate outcomes. For example, shortened G1 phase is associated with accelerated cell-cycle progression and efficient somatic cell reprogramming (Guo et al., 2014). In mouse embryonic stem cells (ESCs), an elongated G1 phase biases the decision toward stem cell self-renewal rather than differentiation (Li et al., 2012; Li and Kirschner, 2014; Calder et al., 2013). On the other hand, in human ESCs, prolonged G1 phase increases the rate of directional differentiation of stem cells (Chetty et al., 2015). These results suggest that cell-cycle remodeling can influence cell fate decisions. Despite these results in cultured cells, it remains to be fully elucidated whether and how cell-cycle remodeling affects cell fate decisions in multicellular organisms.

During cell-cycle progression, the transition from G1 to S phase involves licensing and firing replication origins. Cdc6 is an essential component of the pre-replicative complex (pre-RC) that, along with Orc1-6 and Cdt1, facilitates loading of the replicative helicase MCM2-7 onto DNA in preparation for active DNA synthesis (Bleichert et al., 2017; Costa et al., 2013; Méndez and Stillman, 2003; Bell and Dutta, 2002; Speck et al., 2005; Speck and Stillman, 2007; Randell et al., 2006). The processes of licensing and firing origins in eukaryotic nuclei is heavily influenced by the chromatin features, such as nucleosome densities and post-translational histone modifications (Lipford and Bell, 2001; Patel et al., 2006; Simpson, 1990). Previous studies have shown that nucleosome densities around origins contribute to the formation of the pre-RC and, therefore, the timing and efficiency of firing origins (Lipford and Bell, 2001; Simpson, 1990; McCune et al., 2008; McGuffee et al., 2013; Müller et al., 2014; Raghuraman et al., 1997; Yabuki et al., 2002). While these processes have been extensively characterized for eukaryotes in yeast and cultured cells, it remains unclear how changes in chromatin structure regulate origin licensing and S-phase initiation in multicellular organisms.

**Figure 1.**
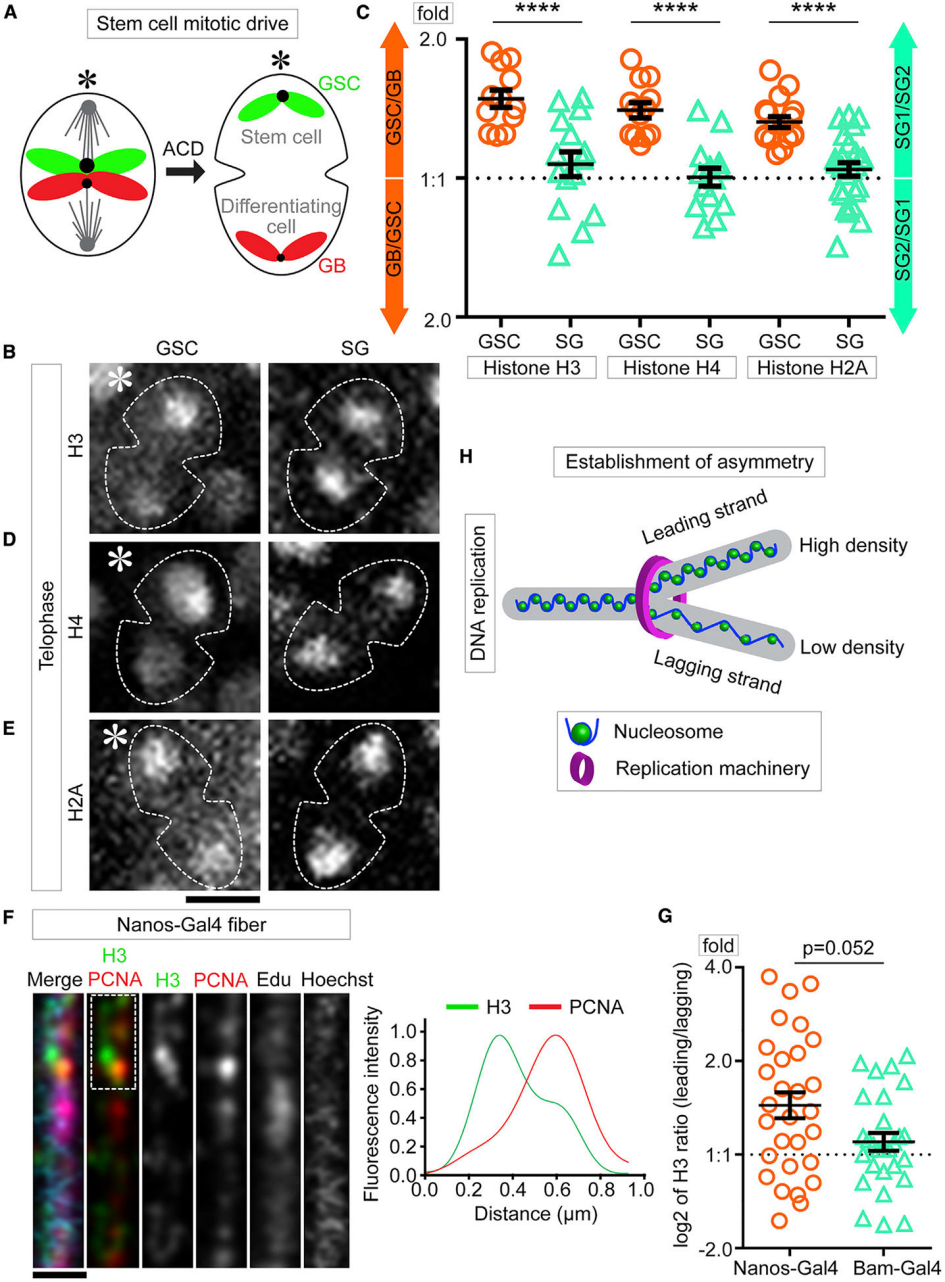
Global nucleosome densities are asymmetric in *Drosophila* male GSCs (A) A cartoon of the asymmetric segregation of old histone (green)- and new histone (red)-enriched sister chromatids in male GSCs. (B, D, and E) Live images for total H3 (B), H4 (D), and H2A (E) for GSCs (left) and SGs (right) at telophase. (C) Quantification of total histone amount by live cell imaging for both GSCs and SGs in telophase (Tables S1–S3, see Mendeley data). H3, GSC 1.50 ± 0.07 (n = 12) and SG 1.10 ± 0.07 (n = 15); H4, GSC 1.42 ± 0.05 (n = 13) and SG 1.02 ± 0.05 (n = 14); H2A, GSC 1.33 ± 0.04 (n = 17) and SG 1.06 ± 0.04 (n = 25), ****p < 10^−4^ by Mann-Whitney t test. (F) Airyscan images of early germline-derived chromatin fiber labeled with H3 (*nanos>H3-GFP*, green), co-labeled with PCNA (red), EdU (magenta), and Hoechst (blue). (G) Quantification of total H3 distribution from both *nanos>H3-GFP* early germline chromatin fibers (log_2_ ratio = 0.53 ± 0.14 [n = 28]) and *bam>H3-GFP* late germline chromatin fibers(log_2_ ratio = 0.14 ± 0.10 [n = 27]∣) (Table S5, see Mendeley data), p = 0.052 by Mann-Whitney t test; p = 0.0007 for *nanos>H3-GFP* fibers by one sample t test and null is log_2_ = 0 for the symmetric pattern; p = 0.1660 for *bam>H3-GFP* fibers by one sample t test and null is log_2_ = 0 for the symmetric pattern. (H) A cartoon shows asymmetric nucleosome density on a replicative chromatin fiber. Scale bars: 5 μm in (B), (D), and (E) and 2 μm in (F). Asterisk: hub. All ratios = average ± SE.

The *Drosophila* germ line provide an excellent model to study how chromatin structure regulates cellular differentiation in adult stem cell lineages (Fuller and Spradling, 2007; Vidaurre and Chen, 2021). Male germline stem cells (GSCs) divide asymmetrically to produce a self-renewing GSC and a differentiating gonialblast (GB) (Yamashita et al., 2003; Kiger et al., 2001; Leatherman and Dinardo, 2010; Tulina and Matunis, 2001). The GB undergoes transit-amplifying divisions as spermatogonial (SG) cells before entering meiosis and terminal differentiation to become mature sperm (Fuller, 1998; White-Cooper et al., 2000). Previously, we have shown that old H3 selectively segregates to the GSC whereas new H3 enriches in the GB during ACD of GSCs (Tran et al., 2012). Mis-regulation of asymmetric histone inheritance results in both GSC loss and progenitor germ cell tumors (Xie et al., 2015). Recently, it was demonstrated that mis-regulation of asymmetric histone inheritance leads to aberrant homologous chromosomal interaction at a key gene required for GSC maintenance, *stat92E*, leading to its abnormal expression (Antel, 2021). Mechanistically, asymmetric histone distribution is established during DNA replication via strand-specific incorporation and biased replication fork movement (Wooten et al., 2019). When the GSC undergoes ACD, epigenetically distinct sister chromatids are differentially recognized and segregated through a “mitotic drive” (Ranjan et al., 2019; Figure 1A). However, questions remain regarding the downstream impacts that epigenetically distinct sister chromatids could have on key molecular and cellular events in the resulting daughter cells.

## RESULTS

### Asymmetric nucleosome density between sister chromatids in GSCs

Chromatin structure affects the binding of key factors that regulate cellular processes such as transcription and DNA replication (Jerković et al., 2020). To better visualize chromatin structure changes in asymmetrically dividing GSCs, we performed high spatial and temporal resolution three-dimensional (3D) live cell imaging using fly lines that express different *histone-eGFP* transgenes controlled by the *nanos-Gal4* driver (Van Doren et al., 1998) in early-stage germ cells, including GSCs (Figure S1A). All live cell imaging was performed without cell cycle arrest, as detailed in (Ranjan and Chen, 2021). When tracking histone inheritance patterns during ACD of GSCs, we found higher levels of histone H3 associate with the sister chromatids segregated toward the future GSC side compared with the sister chromatids segregated toward the future GB side in telophase GSCs (Figure 1B; Video S1; Table S1, see Mendeley Data). Quantification of the entire protein amount showed that ~1.5-fold more H3 was inherited toward the GSC side than the H3 inherited by the GB side (Figures 1C and S1B). In contrast, such an asymmetric H3 was not detected in symmetrically dividing SGs at telophase (Figures 1B, 1C, and S1C; Video S2; Table S1, see Mendeley data). Furthermore, both H4 (Figure 1D; Video S3; Table S2, see Mendeley data) and H2A (Figure 1E; Video S5; Table S3, see Mendeley data) displayed a similar pattern, with ~1.4-fold more H4 and ~1.3-fold more H2A segregating toward the GSC side in telophase GSCs (Figure 1C). Contrastingly, no such asymmetry was detected for either H4 (Video S4) or H2A in telophase SGs (Figure 1C). Both H3 and H2A have variants, such as H3.3, centromere identifier protein (CID) and H2A.v (Ferrand et al., 2020; Henikoff and Smith, 2015; Szenker et al., 2011), but H4 does not have any variant (Holmes et al., 2005; Henikoff and Smith, 2015; Kamakaka and Biggins, 2005). Therefore, the total H4 levels should reliably reflect the genome-wide nucleosome amount.

Next, we utilized a chromatin fiber method coupled with high spatial resolution microscopy to visualize replicative DNA with its associated histones (Wooten et al., 2019, 2020a). First, chromatin fibers were extracted from early-stage germ cells expressing an *H3-eGFP* transgene (*nanos>H3-eGFP*). Regions with active DNA synthesis were labeled with a short pulse of a thymidine analog 5-ethynyl-2′-deoxyuridine (EdU) and imaged using Airyscan microscopy (Sivaguru et al., 2018). Consistent with the asymmetric histone segregation patterns during ACD (Figures 1B and 1C), replicating or newly replicated chromatin fibers displayed an asymmetric distribution of H3-eGFP (Figure 1F). Co-immunolabeling of a lagging strand-enriched protein proliferating cell nuclear antigen (PCNA) (Yu et al., 2014) identified that H3 is more enriched at the PCNA-less leading strand (Figure 1F). When comparing with the ratio between segregated sister chromatids during ACD of GSCs, ~1.44-fold higher nucleosome density was detected at the leading strand relative to the lagging strand (Figure 1G; Table S4, see Mendeley data). The current histone labeling strategy cannot distinguish pure GSC-derived chromatin fibers from other early-stage germ-cell-derived ones, which could underlie the relatively wide distribution of data points (Figure 1G). However, using a late-stage *bag of marbles* driver (*bam-Gal4*) that only turns on transgene expression in late-stage SGs (Eun et al., 2014; Cheng et al., 2008; Chen and McKearin, 2003), *bam>H3-eGFP* fibers showed a significant difference from *nanos>H3-eGFP* fibers but no statistical difference from a symmetric pattern (p = 0.052) (Figure 1G), suggesting that the asymmetric nucleosome density between replicative sister chromatids is more pronounced in early-stage germ cells than in late-stage SGs. To reach an even higher spatial resolution, we performed a super-resolution imaging of chromatin fiber (SRCF) assay using stimulated emission depletion (STED) microscopy (Wooten et al., 2019, 2020a), which showed a similar asymmetric H3 distribution between sister chromatids and similar higher degree of asymmetry of *nanos>H3-eGFP* fibers than *bam>H3-eGFP* fibers (Figures S1D and S1E; Table S5, see Mendeley data). Together, these results indicate that asymmetric nucleosome densities between sister chromatids are likely established during S phase and segregated during M phase in GSCs, with sister chromatids carrying more nucleosomes inherited by the GSCs (Figures 1H and S1F).

### Differential condensation of sister chromatids enriched with old versus new histones

**Figure 2.**
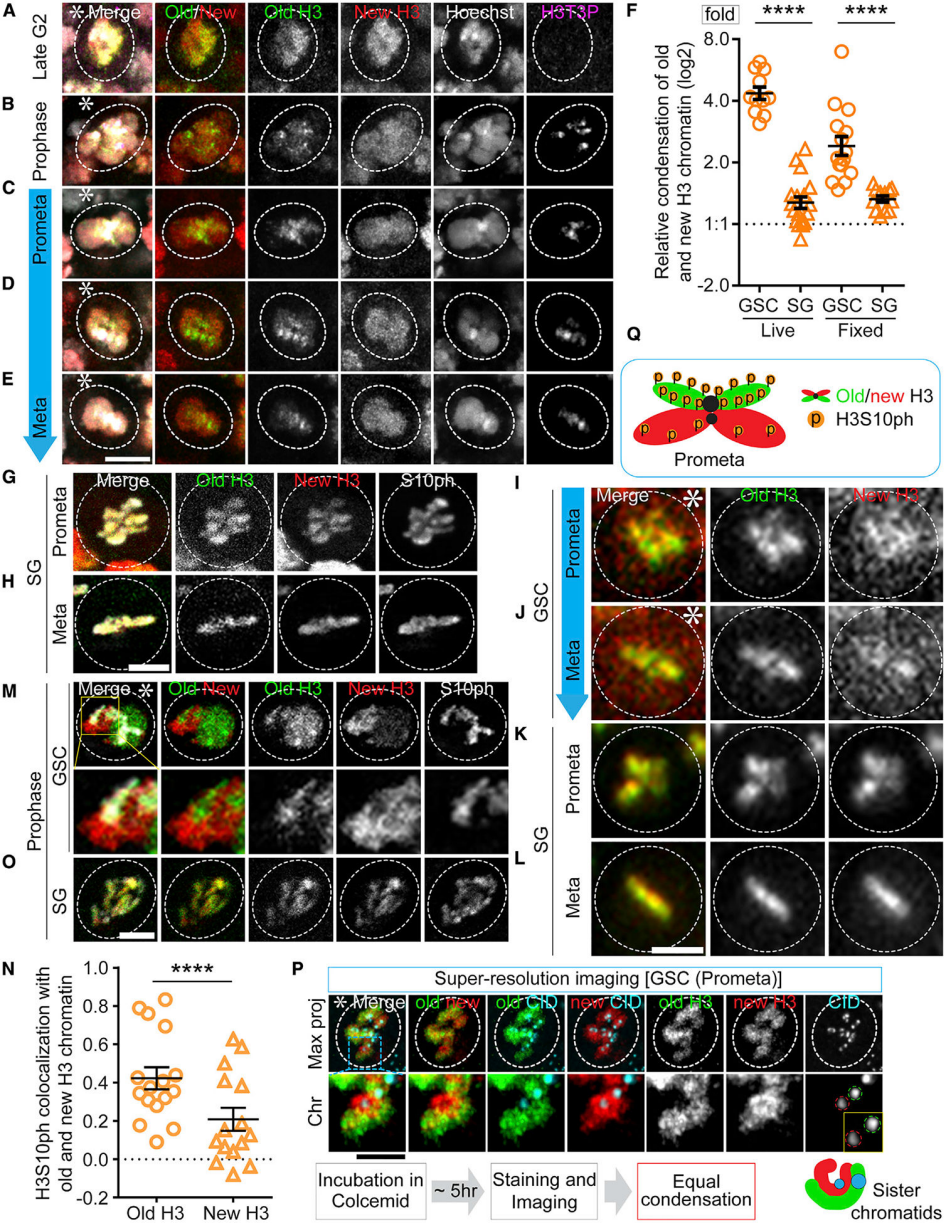
Old H3-enriched chromatin condenses more and earlier than new H3-enriched chromatin in mitotic GSCs (A–E) Fixed cell images of old (eGFP, green) and new (mCherry, new) H3 in GSCs at late G2 phase (A), prophase (B), and prometa-to-metaphase (C–E). A clearance buffer was used to remove free/unbound histones followed by high spatial resolution Airyscan imaging, which showed differential condensation progression in GSCs. (F) Quantification of the relative condensation difference between old and new H3-enriched chromatin in GSCs and SGs, using both live cell and fixed cell imaging (live log_2_ [GSC]: 2.12 ± 0.10 and log_2_ [SG]: 0.35 ± 0.09; fixed log_2_ [GSC]: 1.27 ± 0.15 and log_2_ [SG]: 0.41 ± 0.05; Table S6, see Mendeley data), ****p < 10^−4^ by Mann-Whitney t test. (G–H) Fixed cell images of old and new H3 in SGs at prometaphase (G) and metaphase (H). (I–L) Live cell image snapshots of GSCs at prometa-to-metaphase (I–J) and SGs at prometaphase to metaphase (K and L). (M and O) Airyscan fixed cell images show more association of H3S10P with old H3 than with new H3 in a prophase GSC (M), while equal association of H3S10P with old and new H3 in a prophase SG (O). (N) Spearman correlation coefficient show colocalization of H3S10P with old versus new H3 in GSCs (old H3: 0.49 ± 0.05 and new H3: 0.21 ± 0.06 [n = 16]; Table S7, see Mendeley data), ****p < 10^−4^ by Mann-Whitney t test. (P) Colcemid arrested (~5 h) prometaphase GSCs were imaged with high spatial resolution imaging, showing comparable condensation between old (green) and new (red) histones. Bottom panels show an enlarged chromosomal region from the top panel. Notably, old H3-enriched sister chromatid has a stronger centromere (CID, cyan) than the new H3-enriched sister chromatid, consistent with the previous report (Ranjan et al., 2019). (Q) A cartoon of differential condensation and preferential H3S10P on old H3-enriched sister chromatids in a prometaphase-to-metaphase GSC. Scale bars, 2 μm. Asterisk: hub. All ratios = average ± SE.

Considering this finding of nucleosome density difference between sister chromatids with the previous results that old versus new histone-enriched sister chromatids are established during DNA replication (Wooten et al., 2019) and segregated in mitosis (Ranjan et al., 2019), we hypothesize that the GSC-inherited sister chromatids are enriched with more old histones and have higher nucleosome densities than the GB-inherited ones. To visualize this, we performed high spatial resolution imaging on fixed cells, using a dual-color system to label old H3 with eGFP and new H3 with mCherry (Figure S1A). In mid G2-phase GSCs, old and new H3 showed largely overlapping patterns (Figures 2A and S2C). However, in GSC nuclei from late G2 to M phase, old H3-enriched regions started to display a higher degree of condensation than new H3-enriched domains (Figures 2B-2E, S2A, S2B, and S2D-S2I). Additionally, using a DNA dye we confirmed that the loosely condensed new histone-enriched regions are indeed DNA associated (Figures 2B-2E). Furthermore, using a histone modification enriched at the pericentromeric regions in the mitotic cells, H3 Thr3 phosphorylation (H3T3P or H3T3ph) (Xie et al., 2015), the differentially condensed chromosomal regions are mainly at the separated arms between sister chromatids (Figures 2B-2E). However, old H3- and new H3-enriched regions could condense to a similar degree, if cells were arrested using a microtubule depolymerization drug, colcemid (Venkei and Yamashita, 2015), and given enough time (Figure 2P). In GSCs without any drug treatment, the compaction factor, which is computed as the ratio of new H3-occupied area to old H3-occupied area (Figure S2S), showed an average of 2.64 in M-phase GSCs (Figure 2F), in comparison with 1.35 on average in SGs at the comparable cell-cycle phases (Figures 2F-2H and S2O-S2R). Here, to avoid any possible complication caused by free histones, we used a clearance buffer that has been shown to stringently remove non-chromatin-bound histones in the nucleus (Carroll et al., 2018). Using this strategy, old H3- and new H3-enriched regions still display unequal condensation patterns (Figures 2B-2E). Quantification in the presence and absence of the clearance buffer show comparable compaction factors (Figure S2J). Moreover, this differential condensation is not due to the fluorescence tags for old versus new H3, since swapping the tags to label old H3 with mCherry and new H3 with eGFP yielded similar results (Figures S2K-S2M).

In parallel, high temporal resolution live cell imaging was performed with the dual-color old and new H3 (Figures S2T-S2Y; Video S6). Consistently, old and new H3 largely overlap at mid G2 phase (Figure S2T) but start to display separable patterns from late G2 (Figure S2U) to M phase (Figures 2F, 2I, 2J, and S2V; Video S6) in GSCs. The average compaction factor was 4.46 in GSCs but only 1.32 in SGs at the comparable cell-cycle phases (Figures 2F, 2K, 2L, S2X, and S2Y; Video S7). The higher compaction factor using live cell imaging than using fixed cell imaging (Figure 2F) could be due to an increase of non-chromosome associated new histones in live cells, which are typically washed away in fixed cells (Figure S2J). Together, these data demonstrate that old and new H3-enriched chromatin condense differentially in GSCs but not in SGs.

Based on previous studies, old and new histones carry distinct post-translational modifications. For example, phosphorylation of many residues including both Ser10 (H3S10P or H3S10ph) and H3T3P are enriched on old H3 in human cells using mass spectroscopy (Lin et al., 2016). Here, H3S10P is found to preferentially co-localize with old H3 in GSCs from early prophase to prometaphase (Figures 2M and 2N). By contrast, no such preferential association of H3S10P with old H3 was detected in SGs (Figures 2N and 2O; Table S7, see Mendeley data). These data are consistent with the previous findings that H3T3P is more co-localized with old H3 than with new H3 in GSCs (Xie et al., 2015). Collectively, these results indicate that old H3- and new H3-enriched sister chromatids condense differentially in GSCs, which is at least partially due to the different phosphorylation levels at key H3 residues (Figure 2Q).

### Differential association of a key pre-RC component Cdc6 to sister chromatids in GSCs

These findings raise questions on the biological consequences. As nucleosomes are known to compete with other DNA-binding proteins (Ramachandran and Henikoff, 2016), we hypothesize that these asymmetries could make sister chromatids different substrates for other chromatin-associating factors. For example, condensed chromatin during mitosis provides a binding barrier for a wide variety of proteins (Wang and Higgins, 2013). Thus, the less compact sister chromatid with low nucleosome occupancy could offer a more permissive binding substrate than the sister chromatid with high nucleosome density.

**Figure 3.**
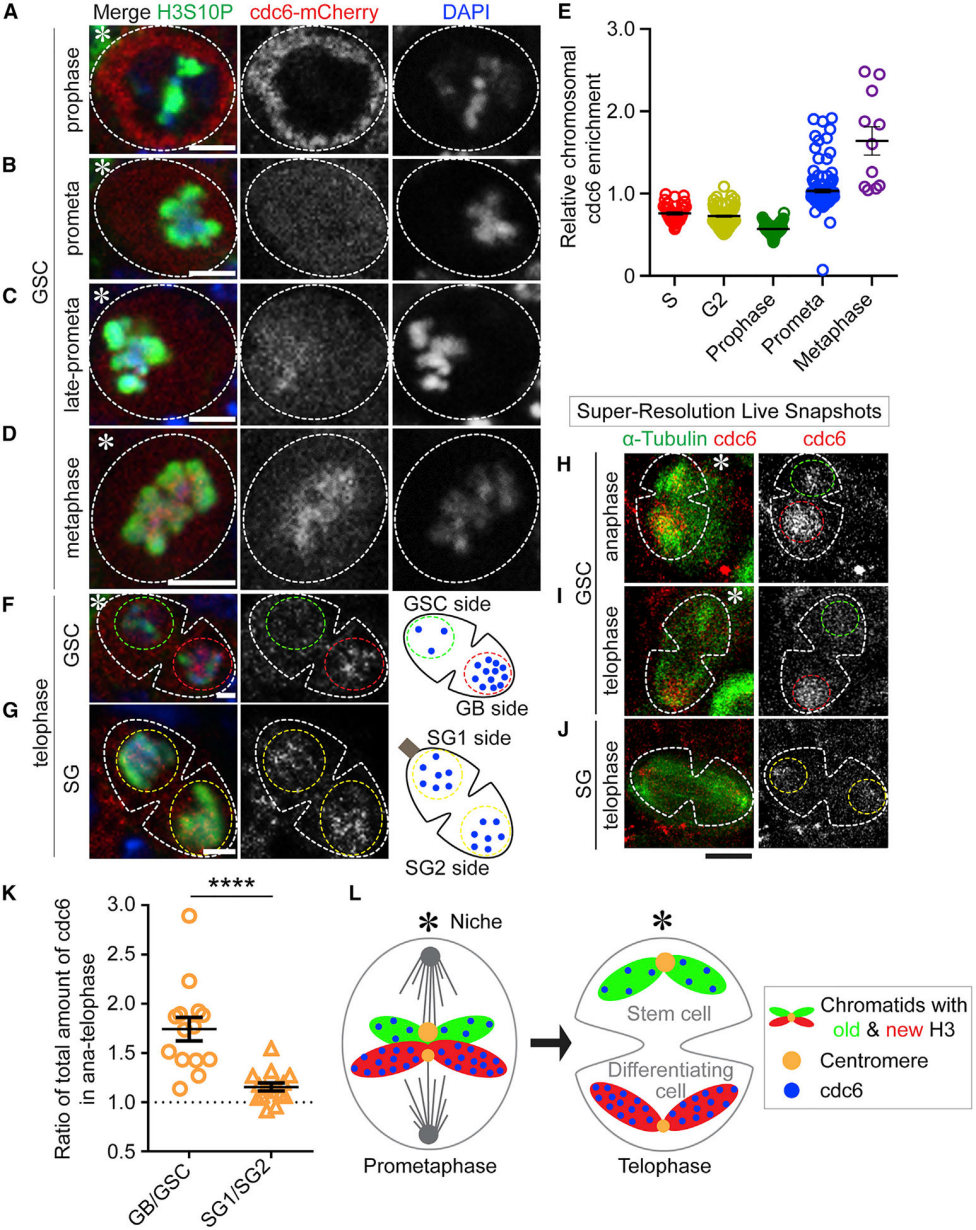
Cdc6 binds to chromatin during mitosis and segregates asymmetrically to the GB during ACD of GSCs (A–D) In GSCs, Cdc6 localizes in the cytoplasm during prophase (A), gradually associates with the mitotic chromatin during prometaphase (B and C), and strongly associates with chromatin at metaphase (D). (E) Quantification of the Cdc6 association with chromatin (see Figure S3A, S phase: 0.76 ± 0.02 [n = 36]; G2 phase: 0.73 ± 0.01 [n = 98]; prophase: 0.57 ± 0.01 [n = 70]; prometaphase: 1.03 ± 0.02 [n = 120]; metaphase: 1.64 ± 0.17 [n = 11]). (F and G) Segregation of Cdc6 during mitosis in GSCs (F) and SGs (G) by fixed cell imaging. (H–J) Airyscan SRLS for Cdc6 segregation pattern in an anaphase GSC (H), a telophase GSC (I), and a telophase SG (J). (K) Distribution of Cdc6 in anaphase/telophase GSCs and SGs (GB/GSC: 1.74 ± 0.12 [n = 14]; SG1/SG2: 1.16 ± 0.04 [n = 15]), ****p < 10^−4^ by Mann-Whitney t test, Table S8, see Mendeley data. (L) A model for the differential binding of Cdc6 to new H3-enriched sister chromatids followed by asymmetric segregation to the GB. Scale bars, 2 μm in (A–D, F–G) and 5 μm in (H–J). Asterisk: hub. All ratios = average ± SE.

We examined this possibility by imaging the mitotic localization of a pre-RC component Cdc6. Since Cdc6 is essential for initiating DNA replication in eukaryotic cells, we generated a fly line with the *mCherry* tag at the endogenous *cdc6* gene, encoding the Cdc6-mCherry fusion protein. We then studied the Cdc6 dynamics by performing high spatial resolution imaging on fixed cells. In GSCs, Cdc6 levels increased from late G2 to M phase but was largely excluded from the prophase nucleus (Figures 3A and 3E). However, the association of Cdc6 with the mitotic chromosomes initiated in prometaphase and increased at metaphase in GSCs (Figures 3B-3E and S3A). In contrast, Cdc6 only associates with chromatin after exiting M phase and entering G1 phase in somatic cells (Paolinelli et al., 2009; Yim and Erikson, 2010). Intriguingly, in telophase GSCs, Cdc6 displayed an asymmetric association with enrichment toward the future GB side (Figure 3F). However, such an asymmetry was not observed in telophase SGs (Figure 3G), even though Cdc6 displayed a similar mitotic chromosome association in SGs (Figures S3B-S3F).

To further validate the dynamics of Cdc6 in live cells, we performed super-resolution live snapshots (SRLS) imaging using Airyscan microscopy (Ranjan and Chen, 2021; Figure S3G). Consistent with the fixed cell imaging data, Cdc6 is excluded from the nucleus until prometaphase, when the levels of chromatin-associated Cdc6 increase (Figure S3H). In GSCs at anaphase and telophase, Cdc6 displays higher levels at the sister chromatids segregated toward the future GB side compared with the GSC side (Figures 3H and 3I). However, in telophase SGs, such an asymmetric Cdc6 pattern was not detected (Figure 3J). Quantification of the SRLS results reveal that ~1.8-fold more Cdc6 associates with the sister chromatids segregated toward the future GB side when compared with the sister chromatids segregated toward the future GSC side in GSCs during ACD (Figure 3K; Table S8, see Mendeley data). Contrastingly, a nearly equal level of Cdc6 was found between the two sets of sister chromatids in symmetrically dividing SGs (Figure 3K; Table S8, see Mendeley data). Collectively, these results demonstrate that Cdc6 differentially binds to sister chromatids and is asymmetrically inherited during ACD of GSCs (Figure 3L).

### Asynchronous initiation of DNA replication in post-mitotic GSC and GB nuclei

**Figure 4.**
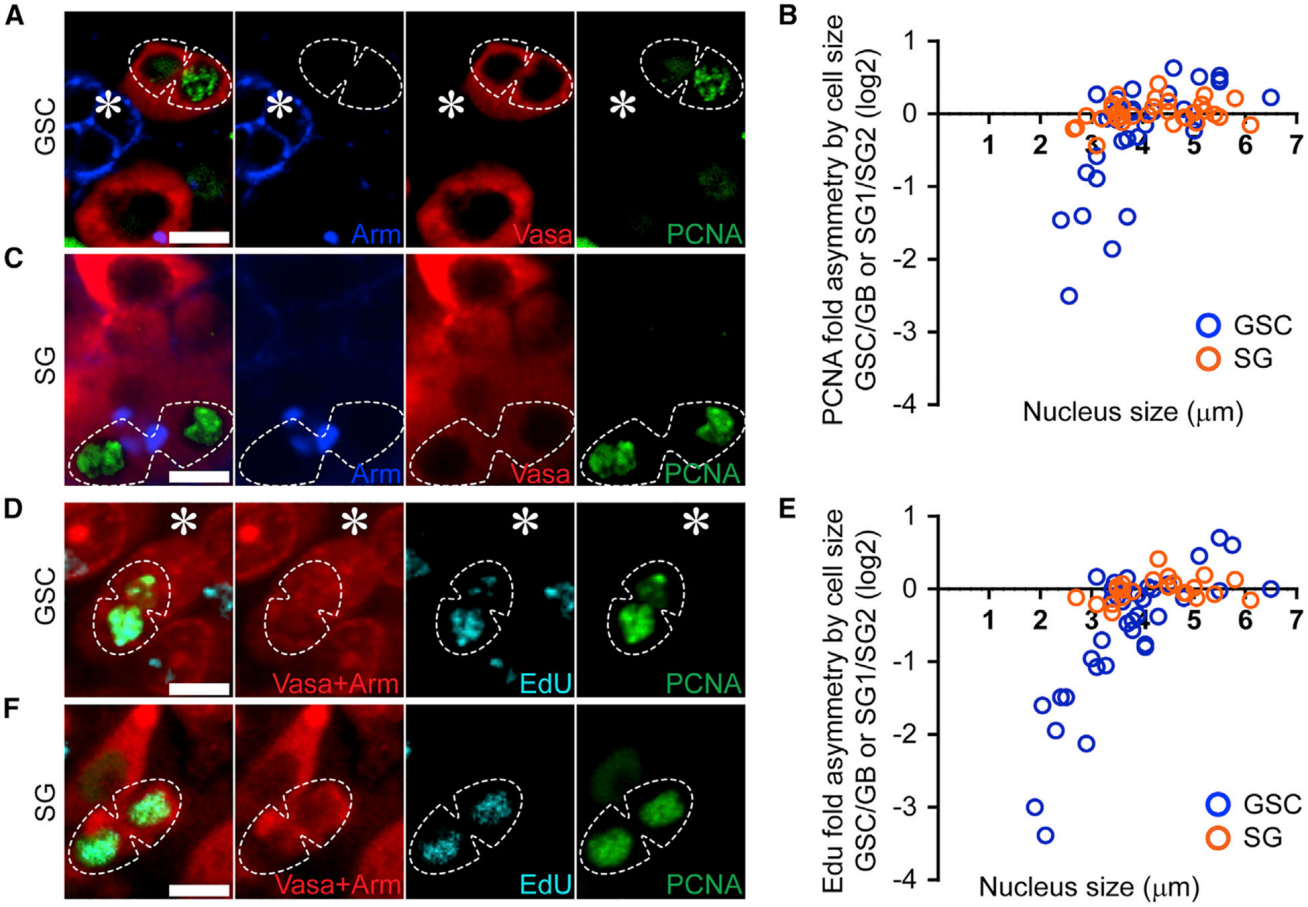
DNA replication initiates a synchronously following ACD of GSCs (A, C, D, and F) PCNA and EdU are enriched in GBs following ACD of GSCs using fixed cell imaging. The GB nucleus is enriched with PCNA (A and D) and EdU (D), whereas the daughter nuclei resulting from a symmetric SG division have comparable levels of PCNA (C and F) and EdU (F). (B and E) Quantification of PCNA (B, n = 33; Table S9, see Mendeley data) and EdU (E, n = 37; Table S10, see Mendeley data) levels in GSC-GB pair nuclei resulting from ACD of GSCs (blue) and SG1-SG2 pair nuclei resulting from symmetric SG divisions (orange), using nuclear size as an indicator of time after mitosis. Scale bars, 2 μm. Asterisk: hub.

Given the essential role of Cdc6 in regulating origin licensing and S-phase initiation (Hossain and Stillman, 2016), we next sought to understand the impact of asymmetric Cdc6 inheritance in the resulting GSC and GB nuclei, respectively. We first investigated PCNA, a replication machinery component critical for DNA polymerase processivity. In ACD-derived GSC-GB pairs, a higher PCNA signal was detected in the GB nucleus compared with the sibling GSC nucleus, indicating that the GB initiates DNA replication prior to the daughter GSC (Figure 4A). Following mitosis, chromosomal decondensation coincides with S-phase progression. Therefore, chromosomal regions or nuclear sizes approximate the relative extent of progression into the subsequent S phase. Quantification of the PCNA ratio in GSC-GB pairs showed that cells with small nuclear sizes indicative of very early S phase displayed predominantly more PCNA in the GB nucleus than in the GSC nucleus (Figures 4B and S4A-S4D; Table S9, see Mendeley data). Cells that progress further into S phase, as indicated by their increased nuclear sizes, showed more comparable PCNA levels in the GSC-GB pair nuclei (Figure 4B), consistent with the previous reports that bulk DNA syntheses are simultaneously detected in GSC-GB pairs derived from ACD of GSCs (Yadlapalli and Yamashita, 2013; Yadlapalli et al., 2011; Sheng and Matunis, 2011; Xie et al., 2015). An asymmetric PCNA distribution was not detected in the symmetrically dividing SG nuclei, using similar assays and criteria (Figures 4B, 4C, and S4A-S4D).

To verify that the PCNA asymmetry reflects asynchronous S-phase initiation, a short pulse of EdU was used to label active DNA synthesis in GSC-GB pair nuclei. Consistent with the PCNA patterns, EdU also displayed an asymmetric incorporation in early S-phase GSC-GB, as high EdU in the GB nucleus while low EdU in the sibling GSC nucleus were detected immediately after exiting M phase (Figures 4D, 4E, and S4A-S4D; Table S10, see Mendeley data). Again, such an asymmetric EdU was not detected in the SG-SG pair nuclei (Figures 4E, 4F, and S4A-S4D; Table S10, see Mendeley data). Overall, these results demonstrate that sister chromatids inherited by GSC and GB are prepared differently in the previous GSC cell cycle to ensure asynchronous subsequent cell-cycle progression from M to S phase (Figures S4E and S4F). The GB rapidly enters the next S phase, whereas the GSC shows a more pronounced G1 phase. Consistent with these observations, quantification of cell-cycle length using live cell imaging demonstrated that GSCs have a longer cell cycle (average = 14.85 h; n = 9) when compared with GBs (average = 12.32 h; n = 7).

### Randomized sister chromatid inheritance abrogates asynchronous initiation of DNA replication in GSC-GB pair nuclei

**Figure 5.**
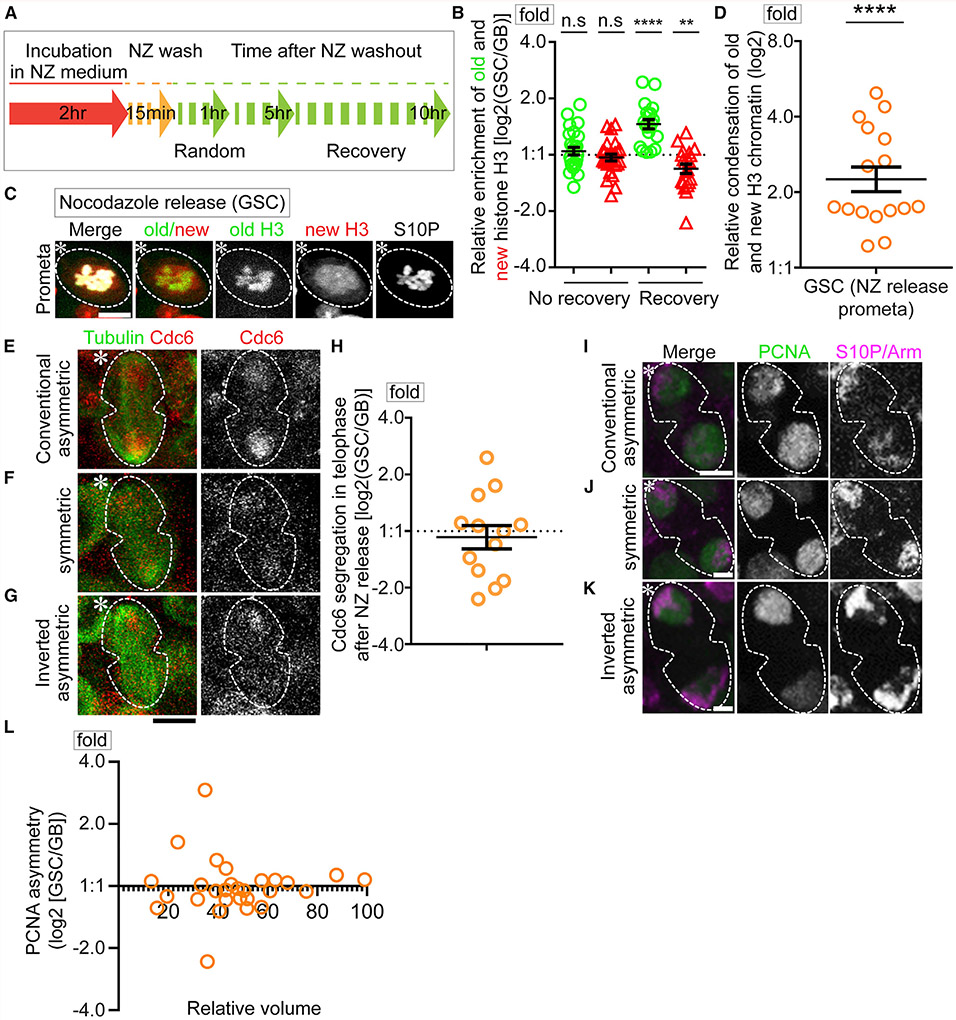
NZ randomizes the asynchronous S-phase entry of the daughter GSC-GB nuclei (A) Scheme of NZ treatment and recovery experiments. (B) Quantification of old and new H3 segregation immediately after release from NZ (no recovery: old H3 log_2_[GSC/GB] = 0.065 ± 0.069; new H3 log_2_[GSC/GB] = −0.048 ± 0.059 [n = 26]; Table S11, see Mendeley data) and after recovering from NZ (recovery: old H3 log_2_[GSC/GB] = 0.544 ± 0.081; new H3 log_2_[GSC/GB] = −0.247 ± 0.083 [n = 20]; Table S11, see Mendeley data), n.s.: not significant, ****p < 10^−4^, **p < 10^−2^ by one sample t test and null is log_2_ = 0 for the symmetric pattern. (C) Differential condensation of old H3 versus new H3 enriched chromatin in a GSC following NZ release, which is comparable with the pattern in the control GSCs without NZ treatment. (D) Quantification of old and new H3 compaction factor with NZ treatment: 2.49 ± 0.30 (n = 16, Table S12, see Mendeley data), ****p < 10^−4^ by one sample t test and null is log_2_ = 0 for equal condensation. (E–G) Airyscan SRLS show randomized Cdc6 patterns with Cdc6 being asymmetrically inherited by the GB (E), symmetrically inherited (F), or asymmetrically inherited by the GSC (G). (H) Quantification of randomized Cdc6 segregation patterns (log_2_[GSC/GB] = −0.109 ± 0.206 [n = 13]; Table S13, see Mendeley data). (I–K) Fixed images of PCNA pattern show that DNA replication initiation is also randomized in post-mitotic pair nuclei with PCNA being asymmetric in the GB (I), symmetric (J), or asymmetric in the GSC (K). (L) Quantification of PCNA levels in GSC-GB pair nuclei resulting from ACD of GSCs, using the relative nuclear volume as an indicator of time after mitosis (n = 28; Table S14, see Mendeley data). Scale bars: 2 μm in (C) and (I)–(K) and 5 μm in (E)–(G). Asterisk: hub. All ratios = average ± SE.

Previously, the microtubule depolymerization drug nocodazole (NZ) was shown to disrupt the temporal asymmetry of microtubules, leading to randomized sister chromatid segregation in male GSCs (Ranjan et al., 2019). Here, we utilized a similar strategy with NZ treatment followed by washout to acutely depolymerize microtubules (Figure 5A). We reason if the asynchronous initiation of DNA replication in GSC-GB pair nuclei is due to the distinct chromatin features between sister chromatids, this treatment should have an impact.

To ensure that the NZ treatment is acute but not persistent, a concern for potential secondary effects, we explored whether the NZ-induced randomized sister chromatid segregation could recover after removing the drug. Using an acute NZ treatment regime (Figure 5A) and live cell imaging, old and new H3 inheritance patterns were found to be mostly randomized immediately after removing NZ (Figures 5B and S5A), consistent with previous results (Ranjan et al., 2019). However, with a recovery after removing NZ, asymmetric H3 inheritance patterns could be restored (Figures 5B and S5B; Table S11, see Mendeley data). These results demonstrate that NZ temporally disrupts asymmetric histone inheritance, but this effect is reversible.

Next, we explored whether NZ treatment disrupts differential sister chromatid condensation (Figure 2), asymmetric Cdc6 inheritance (Figure 3), as well as the asynchronous initiation of DNA replication in the GSC-GB pair nuclei (Figure 4). Immediately after NZ release, old H3- and new H3-enriched regions still displayed differential condensation in prometaphase GSCs, wherein old H3-enriched regions condense 2.49-fold more than new H3-enriched regions (Figures 5C, 5D, and S5C; Table S12, see Mendeley data), comparable to that in untreated GSCs (Figure 2C). These results indicate that the NZ treatment does not interfere with differential condensation of old H3- and new H3-enriched regions, consistent with the idea that the asymmetric incorporation of old and new H3 are established prior to mitosis and recognized by the dynamic microtubules during mitosis, of which only the latter step is disrupted by NZ (Ranjan et al., 2019). However, if Cdc6 preferentially binds to the less condensed new H3-enriched sister chromatids, then NZ treatment should randomize Cdc6 inheritance (Figure S5D). Indeed, live cell imaging showed that Cdc6 segregation is randomized in anaphase and telophase GSCs immediately after NZ release, with asymmetric, symmetric, and inverted asymmetric patterns (Figures 5E-5H; Table S13, see Mendeley data). To further assess potential changes of DNA replication initiation with NZ treatment, PCNA distribution in the GSC-GB pair nuclei was studied using fixed cell imaging. Indeed, NZ treatment results in a randomized PCNA distribution with asymmetric, symmetric, and inverted asymmetric patterns (Figures 5I-5L; Table S14, see Mendeley data).

In summary, the patterns observed in the NZ treated samples can be attributed to the fact that NZ does not disrupt the establishment of histone asymmetries, as has been shown previously for *Drosophila* CID (Ranjan et al., 2019). Rather, NZ abolishes the recognition of sister chromatids bearing distinct chromatin features, resulting in their randomized segregation. As male flies have two sex chromosomes (X and Y) and two major autosomes (2nd and 3rd), even randomized sister chromatid segregation could lead to asymmetric patterns, albeit at a low percentage and without polarity, which were shown previously (Xie et al., 2015). Together, these results indicate that NZ treatment randomizes sister chromatid inheritance, leading to randomized Cdc6 inheritance and asynchronous S-phase entry of the post-mitotic GSC-GB pair.

### The H3S10A mutant abolishes differential sister chromatid condensation and leads to symmetric inheritance of old versus new histones

Since H3S10P preferentially co-localizes with old H3 in GSCs (Figures 2J, 2K, and 2M), and this modification is associated with condensed mitotic chromosome in some contexts (Sawicka and Seiser, 2012; Wei et al., 1998, 1999; Banerjee and Chakravarti, 2011; Hsu et al., 2000; Prigent and Dimitrov, 2003), H3S10P could regulate differential condensation of old H3- versus new H3-enriched sister chromatids in GSCs. To explore this possibility, we generated transgenic fly lines expressing a mutant H3S10A, in which the Ser10 residue was mutated to a non-phosphorylatable alanine (Ala or A). When expressing the H3S10A tagged with eGFP in early-stage germ cells including GSCs (*nanos>H3S10A-eGFP*), the H3S10P signals reduced to ~52.88% of the levels in the control cells expressing wild-type H3 (*nanos>H3-eGFP*, Figures S6A, S6B, and S6D-S6G).

**Figure 6.**
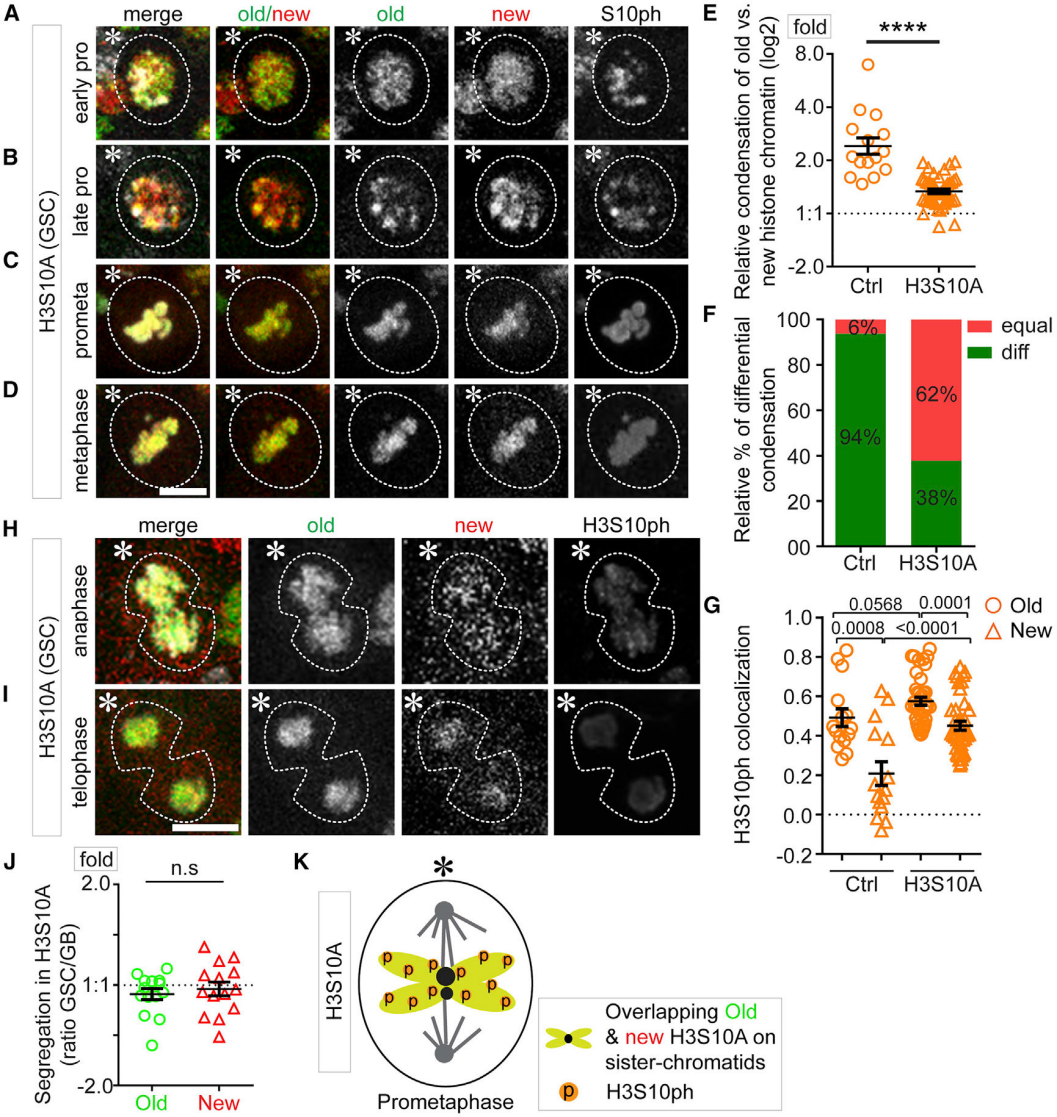
Expression of H3S10A compromises differential condensation and asymmetric inheritance of old versus new H3 (A–D) In GSCs, old and new H3S10A enriched chromatin condense equally in early prophase (A), late prophase (B), prometaphase (C), and metaphase (D). (E) Quantification of the relative condensation between old and new histone enriched chromatin in the control H3- (Ctrl) and H3S10A-expressing GSCs (H3S10A log_2_ [compaction factor] = 0.418 ± 0.043 [n = 45]), ****p < 10^−4^ by Mann-Whitney t test, Table S15, see Mendeley data. (F) The fraction of equally and differentially condensed mitotic sister chromatids in Ctrl and H3S10A-expressing GSCs. (G) Spearman correlation coefficient show colocalization of H3S10P with old versus new histones in Ctrl and H3S10A-expressing GSCs (Ctrl, old H3: 0.49 ± 0.05 and new H3: 0.21 ± 0.06 (n = 16); H3S10A, old H3S10A: 0.58 ± 0.02 and new H3S10A: 0.45 ± 0.02 [n = 41], all p values by Mann-Whitney t test; Table S16, see Mendeley data. (H and I) Old and new H3S10A segregate symmetrically in anaphase and telophase GSCs. (J) Quantification of old and new H3S10A segregation following GSC division (old H3S10A log_2_ [GSC/GB] = −0.090 ± 0.055; new H3S10A log_2_ [GSC/GB] = −0.038 ± 0.068 [n=14]), n.s.: not significant by Mann-Whitney t test, Table S17, see Mendeley data. (K) A model of how H3S10A expression results in comparable H3S10 phosphorylation and sister chromatid condensation in a prometaphase GSC. Scale bars: 2 μm in (A)–(D) and 5 μm in (H) and (I). Asterisk: hub. All ratios = average ± SE.

Using a similar dual-color system to label old H3S10A with eGFP and new H3S10A with mCherry in early-stage germ cells (Figure S1A), we confirmed that the genome-wide incorporation of new H3S10A requires a complete S phase after heat-shock-induced switch from *H3S10A-eGFP-coding* to *H3S10A-mCherry*-coding sequences (Figure S6C), just like wild-type H3 but distinct from the histone variant H3.3 (Kahney et al., 2021; Tran et al., 2012). We then examined and found more overlap between old and new H3S10A signals in GSCs during the second cell cycle after heat shock, from late G2 phase (Figures S6H and S6I) to M phase (Figures 6A-6D, S6J, and S6K). Compared with the 2.64 compaction factor in H3-expressing GSCs, the compaction factor reduced to 1.36 in H3S10A-expressing GSCs (Figure 6E; Table S15, see Mendeley data), comparable to the 1.35 compaction factor in H3-expressing SGs (Figure 2C). Even though some differential condensation could still be detected in H3S10A-expressing GSCs (Figures S6I and S6K), there is a significant decrease of GSCs with differential condensation from 94% in H3-expressing GSCs to 38% in H3S10A-expressing GSCs (Figure 6F). Consistently, the preferential colocalization of H3S10P with old histones was compromised in H3S10A-expressing GSCs, compared with H3-expressing GSCs (Figure 6G; Table S16, see Mendeley data). Together, these data support that phosphorylation at the Ser10 residue of H3 contributes to the differential condensation of old H3- versus new H3-enriched sister chromatids (Figure 2M), which is compromised by the expression of H3S10A.

We next examined whether H3S10A affects asymmetric histone segregation patterns. We observed mostly symmetric old versus new H3S10A segregation in anaphase and telophase GSCs (Figures 6H and 6I). Quantification confirmed that both old H3S10A and new H3S10A displayed nearly symmetric inheritance patterns (Figure 6J; Table S17, see Mendeley data). Notably, these results are different from the NZ-induced randomized inheritance patterns, where asymmetric, symmetric, and inverted asymmetric patterns were all detected (Ranjan et al., 2019), suggesting that H3S10A abolishes the establishment of histone asymmetry prior to mitosis. Collectively, these results indicate that the differential phosphorylation of old and new H3 at Ser10 is required for asymmetric H3 inheritance in GSCs and expressing H3S10A results in a symmetric histone inheritance pattern (Figure 6K).

### H3S10A changes asynchronous initiation of DNA replication in post-mitotic GSC-GB pair and results in GSC defects

**Figure 7.**
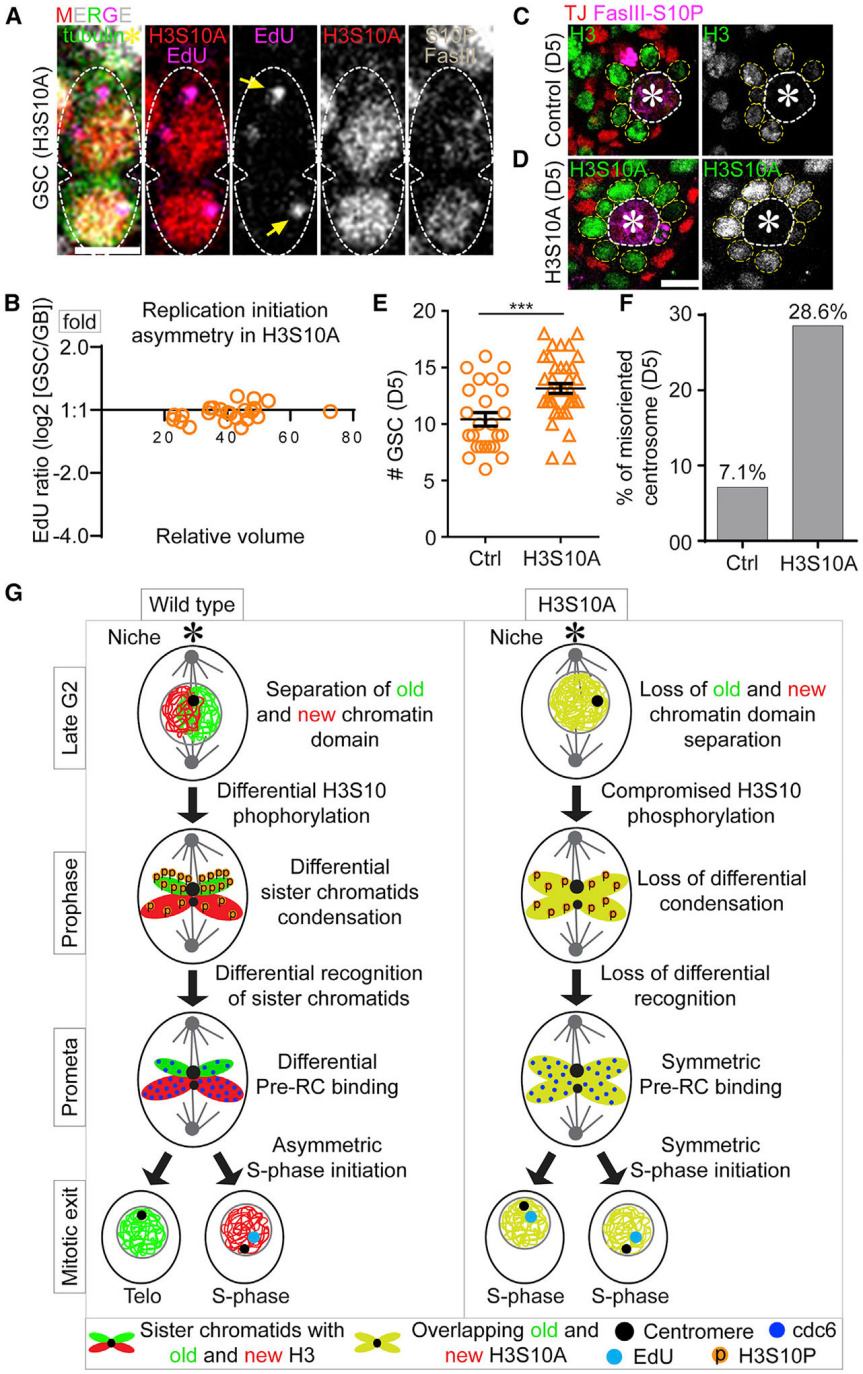
Expression of H3S10A induces synchronous S-phase initiation of GSC-GB pair nuclei and results in several GSC defects (A) Comparable EdU levels and patterns in a late telophase GSC to G1-to-early S phase GSC-GB pair nuclei. (B) Quantification of EdU levels in GSC-GB pair nuclei resulting from ACD of GSCs shows synchronous replication initiation (n = 25, Table S18, see Mendeley data). (C and D) Immunofluorescent images at the testis tip show increased GSCs 5 days (D5) after shifting flies from permissive to restrictive temperature for Gal80^ts^ to turn on *nanos-Gal4* to express H3S10A (D), compared with D5 after expressing the control H3 (C). (E) Quantification of the number of GSCs in testes D5 after expressing either the control H3 or the H3S10A (Ctrl = 10.41 ± 0.51 [n = 29]; H3S10A = 13.16 ±0.44 [n = 38]); ***p = 0.0002, Mann Whitney test. (F) Quantification of the percentage of GSCs with misoriented centrosome D5 after expressing either the control H3 or H3S10A (Ctrl = 7.1% [n = 463]; H3S10A = 28.6% [n = 329]). (G) Models for the role of differential condensation of old and new histone enriched sister chromatids in driving asynchronous S-phase entry of the GSC-GB pair nuclei in wild-type H3- but not in the H3S10A-expressing GSCs. Scale bars, 5 μm. Asterisk: hub. All ratios = average ± SE.

Next, we explored whether H3S10A expression could change the asynchronous S-phase entry in the post-mitotic GSC-GB pair nuclei, using the EdU-pulse labeling method (Figures 4D-4F). Indeed, H3S10A-expressing GSC and GB pairs enter the subsequent S-phase synchronously, shown by the nearly equal EdU levels and similar DNA replication initiation patterns in both daughter nuclei (Figure 7A). Further analyses of the early S-phase GSC-GB pair nuclei, judged by their small nuclear size, revealed nearly equal EdU levels between them (Figure 7B; Table S18, see Mendeley data). Together, these results show nearly synchronous initiation of DNA replication in the H3S10A-expressing GSC-GB pair.

In order to have a precise spatial and temporal control of H3S10A expression, we generated a fly line paring the temperature-sensitive Gal80 driven by the *tubulin* promoter (*tub-Gal80*^*ts*^) with the Gal4 expression controlled by the *nanos* promoter (*tub-Gal80*^*ts*^*; nanos-Gal4>H3S10A-eGFP*). Using this line, we selectively induced H3S10A expression in the early germ line of adult flies. After turning on H3S10A expression for 5 days (D5), we assessed the overall germline fitness and compared it with the control: H3S10A-expressing GSCs showed a decreased mitotic index (1.52%, n = 329) compared with the H3-expressing GSCs (2.38%, n = 463). Interestingly, *nanos>H3S10A* testes showed increased GSCs (13.16/testis, n = 38) compared with the control testes (10.41/testis, n = 29), based on the anatomic position of germ cells surrounding the hub region (Figures 7C-7E and S6E). Furthermore, H3S10A-expressing GSCs showed a 4-fold higher rate of misoriented centrosomes than that of the H3-expressing GSCs (Figure 7F). Together, these results suggest increased dedifferentiation in H3S10A-expressing testes, leading to more GSCs arrested at the G2-M transition due to the “centrosome orientation checkpoint” (Cheng et al., 2008; Venkei and Yamashita, 2015; Yamashita et al., 2007). Consistently, prolonged expression of H3S10A for 10 days (D10) led to decreased GSCs (Figures S7A and S7B), likely due to the long-term consequence of defective GSC cell-cycle progression. Finally, an increased hub area was detected in the H3S10A-expressing testes at D10 (Figure S7C), which has been reported previously as a secondary defect due to GSC loss (Tazuke et al., 2002; Monk et al., 2010; Gönczy and DiNardo, 1996; Dinardo et al., 2011; Tarayrah et al., 2015; Xie et al., 2015; Ranjan et al., 2019). In summary, H3S10A expression disrupts differential condensation of old H3- versus new H3-enriched sister chromatids, leading to defective H3 inheritance and DNA replication initiation in post-mitotic GSC-GB (Figure 7G).

## DISCUSSION

Here, our results provide the direct *in vivo* evidence that the inheritance of epigenetically distinct sister chromatids regulates cell-cycle progression differentially in the daughter cells resulting from ACD. Sister chromatids bearing distinct nucleosome densities can be established, recognized and segregated within one cell cycle but their effects are manifested in the subsequent cell cycle. Such a difference in nucleosome density could significantly impact key chromatin regulators, such as transcription factors and replication proteins, to access and bind to target sites in order to specify cell fates. Disrupting these asymmetries by either abolishing trans-asymmetries such as microtubules using NZ, or *cis*-asymmetries by expressing H3S10A, results in misregulation of the cell-cycle progression and misbehavior of the GSCs (Figure S7D). Together, these results indicate an intimate relationship among chromatin structure, cell-cycle progression, and cell fate decisions.

It has been shown that nucleosome occupancy and position are critical for regulating both transcription and replication, likely due to the fact that histones can directly compete with replication machinery components and transcription factors for binding to key regulatory elements (MacAlpine et al., 2010; Ramachandran and Henikoff, 2016). Histone levels have also been shown to directly regulate transcription factor binding during zygotic genome activation, suggesting nucleosome density modulates DNA accessibility in development (Joseph et al., 2017; Amodeo et al., 2015). Consistently, low nucleosome densities around origins of replication underlie the formation of the pre-RC and facilitate origin firing (Lipford and Bell, 2001; Simpson, 1990; McCune et al., 2008; McGuffee et al., 2013; Müller et al., 2014; Raghuraman et al., 1997; Yabuki et al., 2002). While Cdc6 typically binds to DNA in G1 phase in somatic cells (Paolinelli et al., 2009; Yim and Erikson, 2010), the DNA association of Cdc6 in early-stage *Drosophila* male germ line appears to start in early to mid-M phase. This likely poises the immediate initiation of replication in the GB nucleus without a detectable G1 phase, whereas the GSC nucleus does have a G1 phase and likely has a G1/S checkpoint. Asymmetric Cdc6 association with DNA could promote early S-phase entry through several mechanisms. First, Cdc6 has been well characterized for its role in loading the MCM2-7 helicase during replication licensing (Tanaka et al., 1997; Cook et al., 2002; Yuan et al., 2020). Second, Cdc6 has been found to directly activate *cyclin E* transcription, which is directly responsible for the G1-to-S phase transition (Hossain and Stillman, 2016). Therefore, asymmetric Cdc6 levels could drive accelerated S-phase entry in the GB through loading of the helicase components or/and upregulating *cyclin E* expression.

Previous studies demonstrate that the precise timing of S-phase entry and the tight regulation of G1-phase length shape the trajectory of cellular differentiation. For example, cells at the G1 phase are more sensitive to extrinsic signaling cues than cells at either S- or G2-phase (Pauklin and Vallier, 2013; Gonzales et al., 2015). The timing of exposure to the cues in the G1 phase could significantly affect the outcome of cell fate decisions (Zaveri and Dhawan, 2018). For example, human ESCs exposed to the differentiation cues in early G1 show a higher propensity to become endodermal or mesodermal lineages, whereas human ESCs exposed to differentiation cues in late G1 have a higher likelihood of taking neuro-ectodermal cell fate (Pauklin and Vallier, 2013). These studies suggest that tight regulation of the G1 length could impact how a cell responds to extracellular cues. Here, we propose that the distinct chromatin features in the GB nucleus shorten its G1 length as a means to reduce its opportunity to respond to the niche-emanating signals, which promotes GSC self-renewal. This is critical, as right after ACD of male GSCs, the two daughter GSC and GB are still connected due to delayed cytokinesis and abscission, which could extend to the G1 and S phases and even to the subsequent G2 phase (Lenhart and DiNardo, 2015; Sheng and Matunis, 2011). Therefore, to counteract the prolonged exposure to niche-emanated signals, such as unpaired (Upd) and decapentaplegic (Dpp), the chromatin-regulated truncation of the G1 phase in GB could limit its opportunity to respond to factors that would otherwise prevent differentiation. When asynchronous S-phase entry is disrupted, GBs could regain high sensitivity to the niche-emanated signals, leading to increased GSCs (Figure 7E). In support of this, a significant increase of GSCs with misoriented centrosomes was detected when asynchronous S-phase entry was perturbed (Figure 7F), suggesting that the increased GSCs are derived from GB dedifferentiation, as misoriented centrosomes are often found in dedifferentiated GSC-like cells (Cheng et al., 2008).

In summary, our results demonstrate that the epigenetic differences between sister chromatids contribute to a series of molecular and cellular events that result in distinct remodeling of the subsequent cell cycles in the resulting daughter cells. As cell-cycle progression and timing are inherent properties of each cell type, this work reveals these features as a critical step for cellular differentiation in a stem cell lineage. This work also directly connects asymmetric chromatin to altered cell-cycle progression, prior to gene expression changes in the two distinct daughter cells after mitosis and cytokinesis. We propose a “mitotic cell cycle reprogramming” process that recognizes and executes epigenetically distinct sister chromatids carrying different histones, chromatin statuses, and other chromatin-associated factors for producing distinct daughter cells. In the future, it will be interesting to examine how these features are related to the transcriptome changes (Shi et al., 2020) and how general this mechanism is in other stem cell lineages or during development of multicellular organisms. Moreover, the high spatial and temporal resolution microscopy method could be used to study stem cell properties in the context of the cell cycle at single-cell resolution *in vivo*.

### Limitations of the study

Technically, using chromatin fibers, histone and other chromatin-associated proteins can be visualized at the replicative regions at single-molecule level. However, with the current labeling strategy, early-stage germ cells versus late-stage germ cells can be distinguished but not at single-cell or single differentiation-stage resolution. This complication likely contributes to the relatively wide distribution of data points, which need to be improved with more precise labeling strategy. Moreover, the GSC defects resulted from H3S10A expression was detected a few days after turning on its expression in early-stage germ cells. Therefore, these cellular defects could include both primary and secondary effects. In the future, more direct analyses, such as single-cell chromatin structure and single-cell transcriptome analyses, could be applied to profile more primary “readouts” resulted from misregulation of histone inheritance.

## STAR★METHODS

### RESOURCE AVAILABILITY

#### Lead contact

Further information and requests for resources and reagents should be directed to and will be fulfilled by the lead contact: Xin Chen (xchen32@jhu.edu).

#### Materials availability

- Knock-in fly strains generated in this study is available from the lead contact upon request.

#### Data and code availability

- Original movie and quantification data have been deposited at Mendeley and are publicly available as of the date of publication. The DOI is listed in the key resources table. Microscopy data reported in this paper will be shared by the lead contact upon request.
- This paper does not report original code.
- Any additional information required to reanalyze the data reported in this paper is available from the lead contact upon request.

### EXPERIMENTAL MODEL AND SUBJECT DETAILS

#### Transgenic *Drosophila melanogaster* (fly) strains

Fly stocks were raised using standard Bloomington medium at 18°C, 25°C, or 29°C as noted. The following fly stocks were used: *hs-flp* on the X chromosome (Bloomington Stock Center BL-26902), *nos-Gal4* on the 2nd chromosome (Van Doren et al., 1998), *UASp-FRT-H3-GFP-PolyA-FRT-H3-mKO* on the 3^rd^ chromosome as reported previously (Tran et al., 2012), *UASp-FRT-H3-mCherry -PolyA-FRT-H3-eGFP* on the 3^rd^ chromosome, *UASp-FRT-H4-mCherry -PolyA-FRT-H4-eGFP* on the 3^rd^ chromosome, *UASp-FRT-H2A-mCherry -PolyA-FRT-H2A-eGFP* on the 3^rd^ chromosome, *UAS-α-Tubulin-GFP* (Bloomington Stock Center BL-7373) on the 3^rd^ chromosome, *UASp-FRT-H3S10A-mCherry -PolyA-FRT-H3S10A-eGFP* on the 3^rd^ chromosome, *w; UASp-YFP-PCNA/CyO* (McCleland et al., 2009) (from Patrick O’Farrell, Department of Biochemistry and Biophysics, University of California, San Francisco, USA) and *nos-Gal4 (without VP16)/Cyo; tub-Gal80*^*ts*^*/TM6B* (from Yukiko Yamashita, University of Michigan, Ann Arbor, Michigan, USA). Related to Figures 1, 2, 3, 4, 5, 6, 7, S1-S3, and S5-S7.

#### Generating knock-in *Drosophila melanogaster* (fly) strains

In collaboration with Fungene Inc. (Beijing, China), the following fly lines were generated using the CRISPR-Cas9 technology: CG5971 (*cdc6*) with mCherry tag at the N-terminus, in order to generate the following fusion protein: CDC6-mCherry N term. The mCherry tag does not affect normal function of the *cdc6* gene, as this knock-in line is homozygous viable and has no discernable defect or phenotype. Related to Figures 3, 5, and S3.

### METHOD DETAILS

#### Immunostaining

Immunofluorescence staining was performed using standard procedures (Hime et al., 1996; Tran et al., 2012). Testes from young (0-2 day old) male flies were dissected in 1xPBS buffer. Samples were fixed in 4% formaldehyde in phosphate-buffered saline (PBS) for 10 minutes at room temperature, washed twice, 15 minutes per wash in PBST (PBS with 0.1% Triton X-100), and blocked for at least 30 minutes in PBST with 3% bovine serum albumin at room temperature. Samples were incubated overnight (~16 hours) at 4°C in primary antibodies. Then sample were washed twice, 15 minutes per wash in PBST and incubated in a 1:1,000 dilution of Alexa Fluor-conjugated secondary antibody (Molecular Probes) in PBST for 2 hours at room temperature. Sample were then rinsed quickly three times with PBST, followed by four times 10-minute wash each time with PBST. Samples were mounted for microscopy in Vectashield antifade mounting medium (Vector Laboratories, Cat# H-1400) with or without DAPI, and examined using Leica SPE, Zeiss LSM 700, or Zeiss LSM 800 with Airyscan using the 63× oil immersion objectives. Images of separate fluorochromes from multiple stained tissues were collected individually, combined and analyzed using Fiji and Adobe illustrator (Adobe) software. Related to Figures 2, 3, 4, 5, 6, 7, S1-S3, S6, and S7.

#### Heat shock scheme

Flies with *UASp*-dual color histone transgenes were paired with the *nos-Gal4* driver. Flies were raised at 18°C throughout development until adulthood to avoid pre-flip (Tran et al., 2012). Before heat shock, 1-to-3-day old males were transferred to vials that had been air dried for 24 hours. Vials were submerged underneath water up to the plug in a circulating 37°C water bath for 90 minutes and recovered in a 29°C incubator for indicated time before dissection for immunostaining experiments. Related to Figures 2, 5, 6, S2, S5, and S6.

#### Detection and analysis of the PCNA-YFP signals in testes

Male flies expressing the *UASp-YFP-PCNA* transgene (McCleland et al., 2009) using the *nos-Gal4* driver (Van Doren et al., 1998). Crosses were maintained at 18°C until progenies are eclosed. Progenies were then transferred to new vials and kept at room temperature (RT) for 1-2 days. Testes were then dissected in Schneider’s medium (Gibco, catalog # 21720001) at RT and incubated in Schneider’s medium containing 10μM EdU analog (Invitrogen Click-iT EdU Imaging Kit, catalog # C10340) for 10 minutes, rotating at RT. At the end of the 10 minutes, testes were washed three times with Schneider’s medium at RT. Testes were then fixed, immunostained and mounted as described above. Related to Figures 4 and 5.

#### Chromatin fiber assay and quantification

For a full description of the protocol please refer to Wooten et al. (2020a). In brief, testes were dissected in Schneider’s medium (Gibco, Catalog # 21720001) and then incubated in 10μM EdU for 15 minutes at RT. The Schneider’s medium was then washed off and testes were transferred to lysis buffer (100 mM NaCl, 25 mM Tris-base, 0.2% Joy detergent, pH 10). The testes were then transferred by pipette to a slide with excess lysis buffer. Excess lysis buffer was then removed by Kimwipe and the testis tips were isolated by microdissection to enrich early-stage germ cells. This procedure was done by making an incision at the end of the testis membrane and then allowing the early-stage germ cells to spill out of the tissue before manually removing the rest of the tissue. At the end of the microdissections (~5 min), the lysis buffer solution should nearly dry out. At this point 20μL of lysis buffer was added back to the cells. After 5 minutes, 10μL of a sucrose/formalin (1M sucrose + 10% formaldehyde) mixture was added to the lysis buffer and allowed to sit for another 1 minute. At this point, a 24x60 coverslip (Fisher brand Microscope Cover Glass, 12-545-J 24 × 60 mm) was very slowly set down on top of the solution to spread out fibers. The slide was then moved to −80 °C for 45 min to freeze. After fully freezing, the coverslip was quickly removed by a razor blade and the slide was transferred to 95% cold EtOH for 10 minutes at −20 °C. The slides were then incubated for 1 min in fixative solution (0.5% formaldehyde in 1× PBST). After draining the fixative solution, slides were washed three times in Coplin jars filled with PBST. The slides were then quickly dried and transferred to a humidity chamber with primary antibodies overnight with the slides covered by a small piece of parafilm. After washing three times, the slides were transferred back to the humidity chamber and incubated for two hours with secondary antibodies. For EdU pulse labeling, click chemistry was performed to adhere a 637-Biotin-Azide dye to EdU using a kit (Life Science C10640).

For the total histone amount measurement, the *UASp-FRT-histone-eGFP-PolyA-FRT-H3- mCherry* transgenes were paired with *nanos-Gal4* driver without *hs-flp*. Chromatin fibers were prepared with unflipped histones and stained using the primary antibodies: anti-PCNA(1:200, Santa Cruzsc-56) and anti-GFP (1:1,000; Abcam ab 13970). The slides were then mounted with ProLong Diamond mounting media with DAPI. The fibers were imaged using Airyscan, followed by analyses using FIJI ImageJ by measuring 2μm segments of sister chromatids at replicative regions. The ratios were calculated as log_2_ [(leading strand average intensity – background average intensity)/ (lagging strand average intensity – background average intensity)]. The lagging strand is identified by the enriched PCNA signals. Related to Figures 1 and S1.

#### Live cell imaging

All live cell imaging experiments were performed before cell cycle arrest, as detailed in Ranjan and Chen (2021). To examine the temporal dynamics of cellular processes during asymmetric GSC divisions, we conducted live cell imaging with high temporal resolution (e.g. 1min or 2min interval as mentioned in the supplemental movie legends). To perform live cell imaging, adult *Drosophila* testes were dissected in a medium containing Schneider’s medium with 200μg/ml insulin, 15% (vol/vol) FBS, 0.6x pen/strep, with pH value at approximately 7.0, which we call “live cell medium” as reported previously (Ranjan et al., 2019). Testes were then placed on a Poly-D-lysine coated FluoroDish (World Precision Instrument, Inc.), which contains the live cell medium as described. All movies were taken using spinning disc confocal microscope (Zeiss) equipped with an evolve^-™^ camera (Photometrics), using a 63× objective (1.4 NA) at 29°C. The ZEN 2 software (Zeiss) is used for acquisition with 2x2 binning. Representative videos for live cells are shown in Videos S1, S2, S3, S4, S5, S6, and S7. Related to Figures 1, 2, 5, and S5.

#### Super-Resolution Live Snapshot (SRLS)

To achieve high spatial and temporal resolution imaging to understand the dynamic cellular processes during asymmetric GSC divisions, we conducted Super-Resolution Live Snapshot (SRLS). For a full description of the protocol please see Ranjan and Chen (2021) and Figure S3G. In brief, adult fly testes expressing CDC6-mCherry were dissected in live cell medium (see recipe above). Testes were placed on a Poly-D-lysine coated glass bottom FluoroDish (World Precision Instrument, Inc.) with the live cell medium as described above. Testes were incubated at RT for ~20-30 min before imaging to stabilize the tissue with the *ex vivo* condition and to minimize the interference due to tissue movement. All SRLS was taken using Zeiss LSM 800 confocal microscope with AiryScan module equipped with highly sensitive GaAsP (Gallium Arsenide Phosphide) detectors using a 63× Zeiss objective (1.4 NA) at approximately 20°C. Related to Figures 3, 5, and S3.

#### Fixed cell imaging

To examine the dynamic cellular processes and detailed spatial localization of different cellular components during asymmetric GSC divisions, we conducted fix cell imaging with high spatial resolution. After immunostaining, images were taken using Zeiss LSM 700 confocal microscope, or Zeiss LSM 800 confocal microscope with Airyscan mode with 63× oil immersion objectives. To avoid interference of free histones in the quantification, fixed cell imaging was performed to investigate the differential condensation of old versus new histone-enriched chromatin in control H3-expressing and mutant H3S10A-expressing germ cells at the tip of testis. Images were processed using Imaris software (3D image reconstruction) and Fiji software (to quantify the total amount of protein “RawIntDen” or to generate maximum intensity projection). Related to Figures 2, 3, 4, 5, 6, 7, S1-S3, S6, and S7.

#### Removal of non-chromatin associated proteins with the clearance buffer

Dissect testes at RT in Schneider’s medium. Transfer testes into 1.5ml Eppendorf tube containing 1ml of Schneider’s medium. Prepare the clearance buffer by mixing 989μls of clearance buffer stock solution (8.4 mM HEPES, 100 mM NaCl, 3 mM MgCl, 1 mM EGTA, 300 mM Sucrose, 2% Triton X-1000, and 2% BSA in diH2O) with 1 μl DTT and 10 μl protease inhibitor (100x Leupeptin). Only add the DTT and protease inhibitor immediately before using the buffer. Vortex solution briefly (~10 seconds) to thoroughly mix contents. Remove the Schneider’s medium from the testes and immediately add 1ml of clearance buffer containing DTT and Leupeptin. Rotate the Eppendorf on a nutator for 2-3 minutes at 4°C. While testes are incubating, prepare 1ml of 4% fixative solution by adding 110μl 37% stock solution formaldehyde to 890μl PBST. After 2-3 minutes, remove the sample from the rotator and allow testes to settle to the bottom of the tube. Drain clearance buffer and wash testes 2x with PBST. Add fixative solution and allow testes to rotate on a nutator for 5 mins. Remove the tube from the nutator and allow testes to settle. Drain the fixative solution and wash testes 3x with PBST. After the last wash, incubate testes in 3% BSA blocking buffer for 30 min. While samples are in blocking buffer, prepare primary antibody solution in 3% blocking buffer. Following completion of the 30-min blocking step, drain 3% BSA blocking buffer and immediately add primary antibody solution. Incubate overnight at 4°C on a rotator. Drain primary antibody solution and wash 3x with PBST. After the last wash, add secondary antibody solution (dilution of secondary antibody 1:1000 in blocking buffer); incubate 2 hours at RT or overnight at 4°C on a rotator. Drain secondary antibody solution and wash 3x with PBST before mounting on a slide. Related to Figures 2 and S2.

#### Disruption assays

##### Compromising H3 Serine 10 Phosphorylation by introducing non-phosphorylatable Alanine (serine to alanine)

The *UAS-H3-mCherry* and *UAS-H3S10A-mCherry* flies were crossed with *nanos-Gal4* (*without VP16*)*; tub-Gal80*^*ts*^ flies to generate *tub-Gal80*^*ts*^*, nos-Gal4>H3-mCherry* (*Ctrl*), and *tub-Gal80*^*ts*^*, nos-Gal4> H3S10A-mCherry* (H3S10A mutant) male flies. Crosses were maintained at 18°C (permissive temperature for Gal80^ts^) to keep the Gal4 repressed; therefore, no H3S10A is expressed during development. After eclosion, flies were shifted to 29°C (restrictive temperature for Gal80^ts^) to allow the Gal4 to be active and turn on H3S10A expression for the time as indicated. Related to Figures 6, 7, S6, and S7.

##### Disruption of microtubule asymmetry

Temporal asymmetry in microtubes activity was disrupted using previously standardized conditions in the *Drosophila* male GSCs (Ranjan et al., 2019). To disrupt the temporal asymmetry of microtubules activity at mother and daughter centrosomes, testes were treated with microtubule depolymerizing drug nocodazole (NZ) for 2-3 hours, and NZ was then washed out to allow cells to resume the cell cycle. This disrupts pre-established microtubule asymmetry in GSCs at G2 phase. When cell resume mitosis immediately after washing out NZ, microtubules activity at mother and daughter centrosome becomes nearly symmetric (Ranjan et al., 2019). Here, we considered that when we allow the cell to progress into the next cell cycle after washing out NZ, asymmetric microtubules activity between mother and daughter centrosome could be re-established.

Prior to dissection, NZ solution was freshly prepared by adding 1 μl of 2 mg/ml NZ stock solution in DMSO per 200 μl of “live cell medium” for a final NZ concentration at 10μg/ml. This solution was left in darkness at RT until needed. Testes were dissected in live cell medium and transferred to tubes with excess live cell medium. After removing the live-cell medium, 50μl of NZ solution was added to each tube, which was left open in darkness at RT for 2-4 hours in the NZ solution. At the end of the 2-4 hours, the NZ solution was removed, and tissue was washed using the live cell medium. The medium was removed, and fresh medium was added, repetitively for a total of at least three washes within 5 minutes and incubate the tissue in the live cell medium as required (Figure 5A). For the PCNA and EdU experiments, testes were then fixed approximately 50-60 minutes after release to catch anaphase or telophase cells. For the CDC6 SRLS (Figures 5E-5G) and H3 segregation (Figure 5B) experiments, testes were live imaged immediately after NZ washout, and for the H3 recovery (Figure 5B) experiment, testes were live imaged 4-5 hours after washing out NZ. Related to Figures 5 and S5.

### QUANTIFICATION AND STATISTICAL ANALYSIS

#### The 3D quantification of movies and fix images

To quantify total amount of proteins (such as histones or CDC6) during asymmetric GSC divisions and symmetric SG divisions, we conducted a 3D quantification in volume by measuring the fluorescence signal in each plane from the Z-stack (e.g. Figure S1B) as described in Ranjan et al. (2019). The 3D quantification was done at different cell-cycle stages (as labeled in the corresponding Figures) in GSCs and SGs (e.g. spermatogonial cells from the 8-cell cyst), using time lapse movie with *cdc6-mCherry* knock-in line, and different fluorescence tagged histone lines, such as *H3-mCherry, H4-mCherry*, and *H2A-mCherry* transgenic lines. No antibody was added to enhance the EGFP or mCherry signal for quantification.

The fixed immunostaining images used fluorescence signals of mCherry or EGFP, which tagged histone, PCNA or CDC6, or the thymidine analogue EdU. The live cell images used fluorescence signals of mCherry or EGFP, which tagged histone, PCNA or CDC6.

The 3D quantification of the fluorescence signal was done manually using Fiji (ImageJ). Un-deconvolved raw images as 2D Z-stacks were saved as un-scaled 16-bit TIF images, and the sum of the gray values of pixels in the image (“RawIntDen”) was determined using Fiji (Image J). A circle was drawn to include all fluorescence signals (either EGFP or mCherry), and an identical circle was drawn in the hub region without the *nanos-Gal4* driven transgene expression and is also absent with CDC6-mCherry expression, as the background. The gray values of the fluorescence signal pixels for each Z-stack (Foreground signal, Fs) was calculated by subtracting the gray values of the background signal pixels (Background signal, Bs) from the gray values of the raw signal pixels (Raw signal, Rs). The total amount of the fluorescence signal in the nuclei was calculated by adding the gray values of the fluorescence signal from all Z-stacks. The total amount of the fluorescence signal (Fs) in the nuclei with Z-stacks (Z1+…+Zn): Fs (Z1+…+Zn) = [(Rs-Bs)1+…+(Rs-Bs) n]. The total amount of fluorescence signal or gray value would represent the total amount of protein in the cell (quantification in volume of the cell). This method is also referred to in the text as “the sum of slices method”. Related to Figures 1, 3, 4, 5, 6, and S5.

#### Nucleosome density assay

For nucleosome density quantification, first we calculated the total amount of histone in the nucleus [quantification in volume (3D quantification)], this would represent the amount of histones on the entire genome at that particular cell cycle stage. Two copy of each core histones (2x H3, 2x H4, 2x H2A and 2x H2B) are present in the nucleosome. Therefore, knowing total amount of histone would allow us to calculate global nucleosome density on DNA = ½ amount of histones (Figure S1B). Then, we determined the ratio of nucleosome density between the two sets of sister chromatids at telophase: in GSC by their polarity toward the future GSC side/GB side (GSC/GB, Figure 1A), or in SG by their orientation toward the fusome structure (SG1/SG2, Figure S1C). For global nucleosome density quantification, we used histone H3, H4 and H2A at telophase stage when separated sister chromatids can be clearly visualized. Further, at telophase stage new histone incorporation for the subsequent S phase should not occur yet, therefore nucleosome density could be analyzed accurately between the two sets of sister chromatids.

The nucleosome density ratio (GSC/GB)_telophase_ = (½ total amount of histones toward GSC side/DNA length) / (½ total amount of histones toward GB side/DNA length) = total amount of histones toward GSC side / total amount of histones toward GB side.

We used similar approach to determine nucleosome density difference between the replicative sister chromatids using the chromatin fiber technique. Related to Figures 1 and S1.

#### A quantitative assay for sister chromatids condensation

Compaction levels were quantified using the standard method in the field, which normalizes signal intensity variations that could arise from differences among cell and tissue types, as well as the microscope settings (Maddox et al., 2006; Ladouceur et al., 2017). To quantify differential sister chromatids condensation, we used dual-color histone transgene line (Figure S1A), as described earlier (Tran et al., 2012). The GSCs or the SGs expressing both old and new histones were imaged, and all condensation assays were performed using FIJI (ImageJ) software. A maximum intensity projection was generated for each GSCs or SGs. The largest square region that fit within the GSC or the SG nucleus was cut out for analysis (Figure S2S). Next, the intensity of each pixel of the image was determined using "display_pixel_values" plugins using FIJI (ImageJ) software. Then, the images of each GSC or SG nucleus were individually scaled, setting the minimum intensity to 0 and the maximum to 65,535 (16-bit range). Individual scaling of the image ensures that the fluorescence intensity distribution is independent of fluctuations due to changes in illumination intensity, variations among samples, or photobleaching.

Sister chromatids condensation is accompanied by a progressive change in the shape of the fluorescence intensity distribution of the nuclear histone-EGFP and histone-mCherry signals (Figure S2S). Prior to the mitosis onset and chromatin condensation (such as in interphase), the old histone-EGFP and new histone-mCherry fluorescence signal are relatively homogeneous. When the sister chromatids start to condense as the cell enters mitosis, the old histone-EGFP and new histone-mCherry fluorescence concentrate into a smaller area of the image. To quantify this shift in the fluorescence intensity distribution and the difference in old versus new histone enriched chromatin condensation dynamics, we monitored the pixels across the image with a threshold at 35% of the maximum intensity of the image (scaled intensities: 22,937). Condensation kinetic profiles to compare old histone-enriched chromatids and new histone-enriched chromatids were generated by plotting the percentage of pixels below the threshold (the condensation parameter). For every condition (control or perturbation), the condensation parameters measured for the prometaphase GSCs or SGs were averaged and plotted. Averaging minimizes the contribution of spatial fluctuations. Related to Figures 2, 5, 6, and S2.

#### Quantification of the association between Cdc6 and the mitotic chromosomes

To enrich for prometaphase and metaphase cells, flies were treated with 4μg/mL colcemid for 4hrs after dissection in imaging medium with insulin and left at RT in the darkness. Afterwards, standard immunofluorescent labeling was performed. In FIJI, use a single slice of the cell encapsulating the largest volume of the chromatin region, outline the chromatin region using DAPI signal (Figure S3A). We then measured the average intensity and the area of Cdc6 in this region. We repeated this process to measure the area and Cdc6 intensity throughout the entire slice of the cell. The relative amount of Cdc6 at the chromatin region is calculated by multiplying the area of the chromatin region by the average intensity of Cdc6 present in that region. The relative amount of Cdc6 excluded from the chromatin region is calculated by multiplying the area of the entire cell by the average intensity of Cdc6 throughout the cell and then subtracting the relative amount of Cdc6 at the chromatin region. To obtain a ratio of these amount and therefore the relative portion of Cdc6 that is chromatin bound, simply divide the relative chromatin occupied amount by the relative chromatin excluded amount. This analysis is done using a single slice as it can be unfeasible to define the upper and lower boundaries of the cell membrane. This does mean that this ratio could be a fraction of the target protein (i.e. Cdc6) in that slice associated with chromatin rather than the real fraction of this protein associated with DNA. However, this approach should represent a series of quantitative snapshots for the changes of the target protein’s association with the mitotic chromatin over time. Related to Figures 3, 5, and S3.

#### Analysis of the post-mitotic pairs immediately after mitosis

All imaging analysis and quantification were performed using the imaging processing software FIJI. Post-mitotic GSC-GB pairs were identified by their stereotypical position in perpendicular to the hub resulted from oriented division plane of the GSCs (Yamashita et al., 2003), their temporarily shared cytoplasm and/or the presence of the spectrosome structure between GSC and GB (Tran et al., 2012). The timing after mitosis was approximated using the nuclear size. The nucleus of a cell is maximally compacted at telophase and gradually decompacts in G1/S phase. Nuclear size continues to increase throughout S-phase as DNA content increases. Therefore, the earliest post-mitotic cells are with markedly smaller nucleus than cells in mid-S phase and late-S phase. As such, nucleus size can be used as a measure of time after mitosis. For single slice measurements (Figures 4B and 4E), we assessed nuclear size by calculating the maximum area occupied by a nucleus in a single slice from a z-stack of the entire nucleus. For each slice of the z-stack image, area was assessed using FIJI by drawing a circle around the edge of the nuclear/chromatin signals, such as histones. Once the largest slice was identified, the region contained within the circle was then measured for the fluorescence levels of PCNA-YFP and EdU, respectively. For the sum of slices measurement (Figures 5L and 7B), nuclear size is approximated by summing the areas from all slices and corrected by multiplying by the z-stack size. Total fluorescence in this context is similarly calculated by summing total fluorescence from each slice. For the sum of slice approach with the Nocodazole (NZ) treatment (Figures 5I-5K), a genomic transgene expressing PCNA-EGFP was used (Blythe and Wieschaus, 2016). EdU was measured for the H3S10A disruption assay to be compatible with labeling old and new histones (Figure 7). All values are reported as the ratio of the intensity of PCNA or EdU in the GSC over the GB. Thus, negative values in the earliest cells represent that the GB daughter nucleus enters replication prior to the GSC daughter nucleus. Related to Figures 4, 5, and 7.

#### Mitotic index

Mitotic index was analyzed by using a mitosis-specific modification: histone H3 threonine 3 phosphorylation (H3T3ph). The number of H3T3ph positive GSCs and total GSCs were counted in control testes and testes with perturbations. The percentage of H3T3ph-positive GSCs was calculated for: Mitotic index = H3T3ph-positive GSC# / Total GSC# x 100. Related to Figure 7.

#### Analysis of GSCs with mis-orientated centrosomes

GSCs expressing either the wild-type H3 or the mutant H3S10A were scored. Centrosomes are labeled using anti-γ-Tubulin. In normal GSCs, centrosomes are oriented with the mother centrosome located near the hub-GSC interface, while the daughter centrosome moves toward the opposite side at early G2 phase (Yamashita et al., 2003, 2007). Mother centrosome is anchored by the astral microtubules toward the hub-GSC interface (Yamashita et al., 2007), therefore in normal GSCs one centrosome should always be adjacent to the GSC-hub interface. In GSCs where both centrosomes are positioned away from the GSC-hub interface toward the opposite side, it is defined as misoriented centrosomes (Yamashita et al., 2007). Related to Figure 7.

#### Co-localization assay

The co-localization assay was performed using FIJI (ImageJ) software. The image was imported into ImageJ, and the GSCs or SGs that are in late prophase or prometaphase were selected. We drew a box around GSC or SG of interest using the “box tool” on the toolbar. We used chromatin-bound histone signals to draw the box, and the box should be as tight to the edges of the signals as possible. After drawing the box, the image was duplicated using an option available under the “image” tab on the toolbar (Image>-Duplicate Image). Next, channels were split using an option available under the “image” tab on the toolbar (select Image>Color>Split Channels). Now all channels were available to perform all possible co-localization permutations. To perform co-localization analysis of H3S10ph with old histone versus new histone enriched chromatin regions, Coloc 2 plugin was used (select Analysis>Co-localization>Coloc 2). The Coloc 2 implements and performs the pixel intensity correlation over space (pixel intensity spatial correlation analysis). The analysis was performed on relevant channel combinations such as H3S10P versus old histone H3, H3S10P versus new histone H3. This generates a pdf file, which summarizes the results of the co-localization analysis. We used the Spearman value to compare co-localization between different datasets. The result is +1 for perfect correlation, 0 for no correlation, and −1 for perfect anti-correlation. Related to Figures 2 and S6.

#### Defining different categories of asymmetry

To define different categories (asymmetric and symmetric) for nucleosome density, histone segregation, and CDC6 segregation, we used those ratios in symmetrically dividing SGs (SG1/SG2) to define the symmetric range. For example, H2A nucleosome density in telophase, ratios above mean + SE (1.059 + 0.036 = 1.095) are called ‘asymmetric’ and below are called ‘symmetric’. Related to Figures 1, 3, 5, 6, and S1.

#### Statistics

Statistics analysis was performed in Prism 6 (GraphPad) and was done with Mann-Whitney unpaired t-test and one sample t-test. Data are presented as Average ± SE and significant difference between two groups were noted by asterisks (e.g. **** *P*< 0.0001). Related to Figures 1, 2, 3, 5, 6, 7, S1, S2, and S7.

## Supplementary Material

Supp Info

1

2

3

4

5

6

7

## Figures and Tables

**Table:** KEY RESOURCES TABLE

REAGENT or RESOURCE	SOURCE	IDENTIFIER
Antibodies		
Mouse monoclonal anti- Fasciclin III (Fas III)	Developmental Studies Hybridoma Bank (DSHB)	RRID: AB_528238
Mouse monoclonal anti-alpha Spectrin	Developmental Studies Hybridoma Bank (DSHB)	RRID: AB_528473
Mouse monoclonal anti-Armadillo	Developmental Studies Hybridoma Bank (DSHB)	RRID: AB_528089
Rat monoclonal anti-DE-cadherin	Developmental Studies Hybridoma Bank (DSHB)	RRID: AB_528120
Mouse monoclonal anti-Lamin	Developmental Studies Hybridoma Bank (DSHB)	RRID: AB_528336
Rabbit monoclonal anti-H3T3ph	Millipore	RRID: AB_05-746R
Rabbit anti-Vasa	Santa Cruz	RRID: AB_SC-30210
Mouse monoclonal anti-H3S10ph	Abcam	RRID: AB_14995
Mouse monoclonal anti-γ-tubulin	Sigma Aldrich	RRID: AB_T6557
Guinea pig anti-Traffic jam	Kindly provided by Mark Van Doren, Johns Hopkins University, MD, USA	N/A
Mouse anti-PCNA	Santa Cruz	RRID: AB_SC-56
Chicken anti-GFP	Abcam	RRID: AB_13970
Chemicals, peptides, and recombinant proteins		
FBS	Sigma	Cat#F2442
Penicillin/Streptomycin	Sigma	Cat#P0781
Nocodazole	Sigma	Cat#M1404
Colcemid	Sigma	10295892001 Roche
DMSO	Sigma	Cat#D2650
Mounting Medium	Vector	Cat# H-1400
BSA	Cell Signaling Technology	Cat#9998
Formaldehyde	Fisher Scientific	Cat#F79-500
Schneider Medium	ThermoFisher Scientific	Cat# 21720024
Insulin	ThermoFisher Scientific	Cat#12585014
FluoroDish	World Precision Instrument, Inc.	Cat#FD35PDL
Deposited data		
Supplemental Data	Original/source data for [supplemental movies and tables]	Mendeley Data: https://data.mendeley.com/datasets/znzrnd8zhf/1
Experimental models: Organisms/strains		
*D. melanogaster*. UAS-α-tubulin-GFP	Bloomington Drosophila Stock Center	RRID: BDSC_7373
*D. melanogaster*. hs-flp	Bloomington Drosophila Stock Center	RRID: BDSC_26902
*D. melanogaster*. Nos-Gal4	Kindly provided by Mark Van Doren, Johns Hopkins University, MD, USA	N/A
*D. melanogaster*. Nos-Gal4; tubulin-Gal80^ts^	Kindly provided by Yukiko Yamashita, Whitehead Institute, MIT, USA	N/A
*D. melanogaster*. UAS-H3-mCherry-H3-EGFP or UAS-H3- EGFP-H3-mCherry	Our lab has developed	N/A
*D. melanogaster*. UAS-H4-mCherry-H4-EGFP	Our lab has developed	N/A
*D. melanogaster*. UAS-H2A-mCherry-H2A-EGFP	Our lab has developed	N/A
UASp-YFP-PCNA	Kindly provided by Patrick O’Farrell, UCSF, San Francisco,	N/A
pcna>PCNA-eGFP	Kindly provided by Shelby Blythe and Eric Wieschaus	N/A
*D. melanogaster*. Cdc6-mCherry	Our lab has developed	N/A
Software and algorithms		
Adobe Illustrator CS6	Adobe	N/A
Prism 6	Graphpad	N/A
Fiji	NIH	N/A
EndNote	Clarivate Analytics	N/A
IMARIS	Bitplane	N/A

## Highlights

- Sister chromatids differ in nucleosome density from DNA replication to mitosis
- Sister chromatids undergo differential condensation in mitotic germline stem cells
- Asymmetric association of Cdc6 leads to asynchronous initiation of the next S phase
- Disrupting asymmetric histone inheritance results in cell cycle and cell fate defects

## References

[R1] Allis CD, and Jenuwein T (2016). The molecular hallmarks of epigenetic control. Nat. Rev. Genet 17, 487–500. 27346641 10.1038/nrg.2016.59

[R2] Allshire RC, and Madhani HD (2018). Ten principles of heterochromatin formation and function. Nat. Rev. Mol. Cell Biol 19, 229–244. 29235574 10.1038/nrm.2017.119 PMC6822695

[R3] Amodeo AA, Jukam D, Straight AF, and Skotheim JM (2015). Histone titration against the genome sets the DNA-to-cytoplasm threshold for the Xenopus midblastula transition. Proc. Natl. Acad. Sci. USA 112, E1086–E1095. 25713373 10.1073/pnas.1413990112 PMC4364222

[R4] Antel M, Masoud M, Raj R, Pan Z, Li S, Mellone BG, and Inaba M (2021). Interchromosomal interaction of homologous Stat92E alleles regulates transcriptional switch during stem-cell differentiation. Preprint at bioRxiv. 10.1101/2021.11.08.467622. PMC9271046 35810185

[R5] Atlasi Y, and Stunnenberg HG (2017). The interplay of epigenetic marks during stem cell differentiation and development. Nat. Rev. Genet 18, 643–658. 28804139 10.1038/nrg.2017.57

[R6] Avgustinova A, and Benitah SA (2016). Epigenetic control of adult stem cell function. Nat. Rev. Mol. Cell Biol 17, 643–658. 27405257 10.1038/nrm.2016.76

[R7] Banerjee T, and Chakravarti D (2011). A peek into the complex realm of histone phosphorylation. Mol. Cell. Biol 31, 4858–4873. 22006017 10.1128/MCB.05631-11 PMC3233023

[R8] Bannister AJ, and Kouzarides T (2011). Regulation of chromatin by histone modifications. Cell Res. 21, 381–395. 21321607 10.1038/cr.2011.22 PMC3193420

[R9] Bell SP, and Dutta A (2002). DNA replication in eukaryotic cells. Annu. Rev. Biochem 71, 333–374. 12045100 10.1146/annurev.biochem.71.110601.135425

[R10] Belsky JA, Macalpine HK, Lubelsky Y, Hartemink AJ, and Macalpine DM (2015). Genome-wide chromatin footprinting reveals changes in replication origin architecture induced by pre-RC assembly. Genes Dev. 29, 212–224. 25593310 10.1101/gad.247924.114 PMC4298139

[R11] Blanpain C, and Fuchs E (2014). Stem cell plasticity. Plasticity of epithelial stem cells in tissue regeneration. Science 344, 1242281. 24926024 10.1126/science.1242281 PMC4523269

[R12] Bleichert F, Botchan MR, and Berger JM (2017). Mechanisms for initiating cellular DNA replication. Science 355, eaah6317. 28209641 10.1126/science.aah6317

[R13] Blythe SA, and Wieschaus EF (2016). Establishment and maintenance of heritable chromatin structure during early *Drosophila* embryogenesis. Elife 5, e20148. 27879204 10.7554/eLife.20148 PMC5156528

[R14] Calder A, Roth-Albin I, Bhatia S, Pilquil C, Lee JH, Bhatia M, Levadoux-Martin M, Mcnicol J, Russell J, Collins T, and Draper JS (2013). Lengthened G1 phase indicates differentiation status in human embryonic stem cells. Stem Cells Dev. 22, 279–295. 22827698 10.1089/scd.2012.0168

[R15] Carroll TD, Newton IP, Chen Y, Blow JJ, and Näthke I (2018). Lgr5(+) Intestinal stem cells reside in an unlicensed G1 phase. J. Cell Biol 217, 1667–1685. 29599208 10.1083/jcb.201708023 PMC5940300

[R16] Chen D, and Mckearin DM (2003). A discrete transcriptional silencer in the bam gene determines asymmetric division of the *Drosophila* germline stem cell. Development 130, 1159–1170. 12571107 10.1242/dev.00325

[R17] Cheng J, Türkel N, Hemati N, Fuller MT, Hunt AJ, and Yamashita YM (2008). Centrosome misorientation reduces stem cell division during ageing. Nature 456, 599–604. 18923395 10.1038/nature07386 PMC2712891

[R18] Chetty S, Engquist EN, Mehanna E, Lui KO, Tsankov AM, and Melton DA (2015). A Src inhibitor regulates the cell cycle of human pluripotent stem cells and improves directed differentiation. J. Cell Biol 210, 1257–1268. 26416968 10.1083/jcb.201502035 PMC4586752

[R19] Cook JG, Park CH, Burke TW, Leone G, Degregori J, Engel A, and Nevins JR (2002). Analysis of Cdc6 function in the assembly of mammalian prereplication complexes. Proc. Natl. Acad. Sci. USA 99, 1347–1352. 11805305 10.1073/pnas.032677499 PMC122193

[R20] Costa A, Hood IV, and Berger JM (2013). Mechanisms for initiating cellular DNA replication. Annu. Rev. Biochem 82, 25–54. 23746253 10.1146/annurev-biochem-052610-094414 PMC4696014

[R21] Dalton S (2015). Linking the cell cycle to cell fate decisions. Trends Cell Biol. 25, 592–600. 26410405 10.1016/j.tcb.2015.07.007 PMC4584407

[R22] Dinardo S, Okegbe T, Wingert L, Freilich S, and Terry N (2011). lines and bowl affect the specification of cyst stem cells and niche cells in the *Drosophila* testis. Development 138, 1687–1696. 21486923 10.1242/dev.057364 PMC3074445

[R23] Eun SH, Shi Z, Cui K, Zhao K, and Chen X (2014). A non-cell autonomous role of E(z) to prevent germ cells from turning on a somatic cell marker. Science 343, 1513–1516. 24675960 10.1126/science.1246514 PMC4040133

[R24] Feng L, and Chen X (2015). Epigenetic regulation of germ cells-remember or forget? Curr. Opin. Genet. Dev 31, 20–27. 25930104 10.1016/j.gde.2015.04.003 PMC4470759

[R25] Ferrand J, Rondinelli B, and Polo SE (2020). Histone variants: guardians of genome integrity. Cells 9, 2424. 10.3390/cells9112424 PMC7694513 33167489

[R26] Fuller MT (1998). Genetic control of cell proliferation and differentiation in *Drosophila* spermatogenesis. Semin. Cell Dev. Biol 9, 433–444. 9813190 10.1006/scdb.1998.0227

[R27] Fuller MT, and Spradling AC (2007). Male and female *Drosophila* germline stem cells: two versions of immortality. Science 316, 402–404. 17446390 10.1126/science.1140861

[R28] Tarayrah L, Li Y, Gan Q, and Chen X (2015). Epigenetic regulator Lid maintains germline stem cells through regulating JAK-STAT signaling pathway activity. Biol. Open 4, 1518–1527. 26490676 10.1242/bio.013961 PMC4728359

[R29] Goldberg AD, Allis CD, and Bernstein E (2007). Epigenetics: a landscape takes shape. Cell 128, 635–638. 17320500 10.1016/j.cell.2007.02.006

[R30] Gönczy P, and Dinardo S (1996). The germ line regulates somatic cyst cell proliferation and fate during *Drosophila* spermatogenesis. Development 122, 2437–2447. 8756289 10.1242/dev.122.8.2437

[R31] Gonzales KA, Liang H, Lim YS, Chan YS, Yeo JC, Tan CP, Gao B, Le B, Tan ZY, Low KY, (2015). Deterministic restriction on pluripotent state dissolution by cell-cycle pathways. Cell 162, 564–579. 26232226 10.1016/j.cell.2015.07.001

[R32] Guo S, Zi X, Schulz VP, Cheng J, Zhong M, Koochaki SH, Megyola CM, Pan X, Heydari K, Weissman SM, (2014). Nonstochastic reprogramming from a privileged somatic cell state. Cell 156, 649–662. 24486105 10.1016/j.cell.2014.01.020 PMC4318260

[R33] Henikoff S, and Smith MM (2015). Histone variants and epigenetics. Cold Spring Harb. Perspect. Biol 7, a019364. 25561719 10.1101/cshperspect.a019364 PMC4292162

[R34] Hime GR, Brill JA, and Fuller MT (1996). Assembly of ring canals in the male germ line from structural components of the contractile ring. J. Cell Sci 109, 2779–2788. 9013326 10.1242/jcs.109.12.2779

[R35] Holmes WF, Braastad CD, Mitra P, Hampe C, Doenecke D, Albig W, Stein JL, Van Wijnen AJ, and Stein GS (2005). Coordinate control and selective expression of the full complement of replication-dependent histone H4 genes in normal and cancer cells. J. Biol. Chem 280, 37400–37407. 16131487 10.1074/jbc.M506995200

[R36] Hossain M, and Stillman B (2016). Opposing roles for DNA replication initiator proteins ORC1 and CDC6 in control of cyclin E gene transcription. ELife 5, e12785. 27458800 10.7554/eLife.12785 PMC4987141

[R37] Hsu JY, Sun ZW, Li X, Reuben M, Tatchell K, Bishop DK, Grushcow JM, Brame CJ, Caldwell JA, Hunt DF, (2000). Mitotic phosphorylation of histone H3 is governed by Ipl1/aurora kinase and Glc7/PP1 phosphatase in budding yeast and nematodes. Cell 102, 279–291. 10975519 10.1016/s0092-8674(00)00034-9

[R38] Hu X, Eastman AE, and Guo S (2019). Cell cycle dynamics in the reprogramming of cellular identity. FEBS Lett. 593, 2840–2852. 31562821 10.1002/1873-3468.13625

[R39] Jerković I, Szabo Q, Bantignies F, and Cavalli G (2020). Higher-order chromosomal structures mediate genome function. J. Mol. Biol 432, 676–681. 31689436 10.1016/j.jmb.2019.10.014

[R40] Jiang C, and Pugh BF (2009). Nucleosome positioning and gene regulation: advances through genomics. Nat. Rev. Genet 10, 161–172. 19204718 10.1038/nrg2522 PMC4860946

[R41] Joseph SR, Pálfy M, Hilbert L, Kumar M, Karschau J, Zaburdaev V, Shevchenko A, and Vastenhouw NL (2017). Competition between histone and transcription factor binding regulates the onset of transcription in zebra-fish embryos. Elife 6, e23326. 28425915 10.7554/eLife.23326 PMC5451213

[R42] Kahney EW, Zion EH, Sohn L, Viets-Layng K, Johnston R, and Chen X (2021). Characterization of histone inheritance patterns in the *Drosophila* female germline. EMBO Rep. 22, e51530. 34031963 10.15252/embr.202051530 PMC8406404

[R43] Kamakaka RT, and Biggins S (2005). Histone variants: deviants? Genes Dev. 19, 295–310. 15687254 10.1101/gad.1272805

[R44] Kiger AA, Jones DL, Schulz C, Rogers MB, and Fuller MT (2001). Stem cell self-renewal specified by JAK-STAT activation in response to a support cell cue. Science 294, 2542–2545. 11752574 10.1126/science.1066707

[R45] Kornberg RD (1974). Chromatin structure: a repeating unit of histones and DNA. Science 184, 868–871. 4825889 10.1126/science.184.4139.868

[R46] Kornberg RD, and Lorch Y (2020). Primary role of the nucleosome. Mol. Cell 79, 371–375. 32763226 10.1016/j.molcel.2020.07.020

[R47] Ladouceur AM, Ranjan R, Smith L, Fadero T, Heppert J, Goldstein B, Maddox AS, and Maddox PS (2017). CENP-A and topoisomerase-II antagonistically affect chromosome length. J. Cell Biol 216, 2645–2655. 28733327 10.1083/jcb.201608084 PMC5584148

[R48] Lai WKM, and Pugh BF (2017). Understanding nucleosome dynamics and their links to gene expression and DNA replication. Nat. Rev. Mol. Cell Biol 18, 548–562. 28537572 10.1038/nrm.2017.47 PMC5831138

[R49] Leatherman JL, and Dinardo S (2010). Germline self-renewal requires cyst stem cells and stat regulates niche adhesion in *Drosophila* testes. Nat. Cell Biol 12, 806–811. 20622868 10.1038/ncb2086 PMC2917891

[R50] Lenhart KF, and Dinardo S (2015). Somatic cell encystment promotes abscission in germline stem cells following a regulated block in cytokinesis. Dev. Cell 34, 192–205. 26143993 10.1016/j.devcel.2015.05.003 PMC4519359

[R51] Li VC, Ballabeni A, and Kirschner MW (2012). Gap 1 phase length and mouse embryonic stem cell self-renewal. Proc. Natl. Acad. Sci. USA 109, 12550–12555. 22802651 10.1073/pnas.1206740109 PMC3412034

[R52] Li VC, and Kirschner MW (2014). Molecular ties between the cell cycle and differentiation in embryonic stem cells. Proc. Natl. Acad. Sci. USA 111, 9503–9508. 24979803 10.1073/pnas.1408638111 PMC4084474

[R53] Lin S, Yuan ZF, Han Y, Marchione DM, and Garcia BA (2016). Preferential phosphorylation on old histones during early mitosis in human cells. J. Biol. Chem 291, 15342–15357. 27226594 10.1074/jbc.M116.726067 PMC4946945

[R54] Lipford JR, and Bell SP (2001). Nucleosomes positioned by ORC facilitate the initiation of DNA replication. Mol. Cell 7, 21–30. 11172708 10.1016/s1097-2765(01)00151-4

[R55] Lu KL, and Yamashita YM (2017). Germ cell connectivity enhances cell death in response to DNA damage in the *Drosophila* testis. Elife 6, e27960. 28809158 10.7554/eLife.27960 PMC5577909

[R56] Luger K, Mäder AW, Richmond RK, Sargent DF, and Richmond TJ (1997). Crystal structure of the nucleosome core particle at 2.8-A resolution. Nature 389, 251–260. 9305837 10.1038/38444

[R57] Macalpine HK, Gordân R, Powell SK, Hartemink AJ, and Macalpine DM (2010). *Drosophila* ORC localizes to open chromatin and marks sites of cohesin complex loading. Genome Res. 20, 201–211. 19996087 10.1101/gr.097873.109 PMC2813476

[R58] Maddox PS, Portier N, Desai A, and Oegema K (2006). Molecular analysis of mitotic chromosome condensation using a quantitative time-resolved fluorescence microscopy assay. Proc. Natl. Acad. Sci. USA 103, 15097–15102. 17005720 10.1073/pnas.0606993103 PMC1622782

[R59] Mccleland ML, Shermoen AW, and O’Farrell PH (2009). DNA replication times the cell cycle and contributes to the mid-blastula transition in *Drosophila* embryos. J. Cell Biol 187, 7–14. 19786576 10.1083/jcb.200906191 PMC2762091

[R60] Mccune HJ, Danielson LS, Alvino GM, Collingwood D, Delrow JJ, Fangman WL, Brewer BJ, and Raghuraman MK (2008). The temporal program of chromosome replication: genomewide replication in clb5{Delta} *Saccharomyces cerevisiae*. Genetics 180, 1833–1847. 18832352 10.1534/genetics.108.094359 PMC2600925

[R61] Mcguffee SR, Smith DJ, and Whitehouse I (2013). Quantitative, genome-wide analysis of eukaryotic replication initiation and termination. Mol. Cell 50, 123–135. 23562327 10.1016/j.molcel.2013.03.004 PMC3628276

[R62] Méndez J, and Stillman B (2003). Perpetuating the double helix: molecular machines at eukaryotic DNA replication origins. BioEssays 25, 1158–1167. 14635251 10.1002/bies.10370

[R63] Meshorer E, and Misteli T (2006). Chromatin in pluripotent embryonic stem cells and differentiation. Nat. Rev. Mol. Cell Biol 7, 540–546. 16723974 10.1038/nrm1938

[R64] Monk AC, Siddall NA, Volk T, Fraser B, Quinn LM, Mclaughlin EA, and Hime GR (2010). How is required for stem cell maintenance in the *Drosophila* testis and for the onset of transit-amplifying divisions. Cell Stem Cell 6, 348–360. 20362539 10.1016/j.stem.2010.02.016

[R65] Müller CA, Hawkins M, Retkute R, Malla S, Wilson R, Blythe MJ, Nakato R, Komata M, Shirahige K, De Moura AP, and Nieduszynski CA (2014). The dynamics of genome replication using deep sequencing. Nucleic Acids Res. 42, e3. 24089142 10.1093/nar/gkt878 PMC3874191

[R66] Paolinelli R, Mendoza-Maldonado R, Cereseto A, and Giacca M (2009). Acetylation by GCN5 regulates CDC6 phosphorylation in the S phase of the cell cycle. Nat. Struct. Mol. Biol 16, 412–420. 19343071 10.1038/nsmb.1583

[R67] Patel PK, Arcangioli B, Baker SP, Bensimon A, and Rhind N (2006). DNA replication origins fire stochastically in fission yeast. Mol. Biol. Cell 17, 308–316. 16251353 10.1091/mbc.E05-07-0657 PMC1345668

[R68] Pauklin S, and Vallier L (2013). The cell-cycle state of stem cells determines cell fate propensity. Cell 155, 135–147. 24074866 10.1016/j.cell.2013.08.031 PMC3898746

[R69] Prigent C, and Dimitrov S (2003). Phosphorylation of serine 10 in histone H3, what for? J. Cell Sci 116, 3677–3685. 12917355 10.1242/jcs.00735

[R70] Raghuraman MK, Brewer BJ, and Fangman WL (1997). Cell cycle-dependent establishment of a late replication program. Science 276, 806–809. 9115207 10.1126/science.276.5313.806

[R71] Ramachandran S, and Henikoff S (2016). Transcriptional regulators compete with nucleosomes post-replication. Cell 165, 580–592. 27062929 10.1016/j.cell.2016.02.062 PMC4855302

[R72] Randell JC, Bowers JL, Rodríguez HK, and Bell SP (2006). Sequential ATP hydrolysis by Cdc6 and ORC directs loading of the Mcm2-7 helicase. Mol. Cell 21, 29–39. 16387651 10.1016/j.molcel.2005.11.023

[R73] Ranjan R, and Chen X (2021). Super-resolution live cell imaging of subcellular structures. J. Vis. Exp 10.3791/61563. PMC8197282 33522506

[R74] Ranjan R, Snedeker J, and Chen X (2019). Asymmetric centromeres differentially coordinate with mitotic machinery to ensure biased sister chromatid segregation in germline stem cells. Cell Stem Cell 25, 666–681.e5. 31564548 10.1016/j.stem.2019.08.014 PMC6842444

[R75] Rhind N, and Gilbert DM (2013). DNA replication timing. Cold Spring Harb. Perspect. Biol 5, a010132. 23838440 10.1101/cshperspect.a010132 PMC3721284

[R76] Richmond TJ, and Davey CA (2003). The structure of DNA in the nucleosome core. Nature 423, 145–150. 12736678 10.1038/nature01595

[R77] Rodriguez J, Lee L, Lynch B, and Tsukiyama T (2017). Nucleosome occupancy as a novel chromatin parameter for replication origin functions. Genome Res. 27, 269–277. 27895110 10.1101/gr.209940.116 PMC5287232

[R78] Sawicka A, and Seiser C (2012). Histone H3 phosphorylation - a versatile chromatin modification for different occasions. Biochimie 94, 2193–2201. 22564826 10.1016/j.biochi.2012.04.018 PMC3480636

[R79] Sheng XR, and Matunis E (2011). Live imaging of the *Drosophila* spermatogonial stem cell niche reveals novel mechanisms regulating germline stem cell output. Development 138, 3367–3376. 21752931 10.1242/dev.065797 PMC3143561

[R80] Shi Z, Lim C, Tran V, Cui K, Zhao K, and Chen X (2020). Single-cyst transcriptome analysis of *Drosophila* male germline stem cell lineage. Development 147, dev184259. 32122991 10.1242/dev.184259 PMC7174844

[R81] Simpson RT (1990). Nucleosome positioning can affect the function of a cis-acting DNA element *in vivo*. Nature 343, 387–389. 2405281 10.1038/343387a0

[R82] Sivaguru M, Urban MA, Fried G, Wesseln CJ, Mander L, and Punyasena SW (2018). Comparative performance of airyscan and structured illumination superresolution microscopy in the study of the surface texture and 3D shape of pollen. Microsc. Res. Tech 81, 101–114. 27476493 10.1002/jemt.22732

[R83] Soufi A, and Dalton S (2016). CyclinG through developmental decisions: how cell cycle dynamics control pluripotency, differentiation and reprogramming. Development 143, 4301–4311. 27899507 10.1242/dev.142075 PMC5201050

[R84] Speck C, Chen Z, Li H, and Stillman B (2005). ATPase-dependent cooperative binding of ORC and Cdc6 to origin DNA. Nat. Struct. Mol. Biol 12, 965–971. 16228006 10.1038/nsmb1002 PMC2952294

[R85] Speck C, and Stillman B (2007). Cdc6 ATPase activity regulates ORC x Cdc6 stability and the selection of specific DNA sequences as origins of DNA replication. J. Biol. Chem 282, 11705–11714. 17314092 10.1074/jbc.M700399200 PMC3033201

[R86] Stancheva I (2011). Revisiting heterochromatin in embryonic stem cells. PLoS Genet. 7, e1002093. 21655082 10.1371/journal.pgen.1002093 PMC3107196

[R87] Stillman B (2018). Histone modifications: insights into their influence on gene expression. Cell 175, 6–9. 30217360 10.1016/j.cell.2018.08.032

[R88] Struhl K, and Segal E (2013). Determinants of nucleosome positioning. Nat. Struct. Mol. Biol 20, 267–273. 23463311 10.1038/nsmb.2506 PMC3740156

[R89] Sunchu B, and Cabernard C (2020). Principles and mechanisms of asymmetric cell division. Development 147, dev167650. 32601056 10.1242/dev.167650 PMC7338270

[R90] Szenker E, Ray-Gallet D, and Almouzni G (2011). The double face of the histone variant H3.3. Cell Res. 21, 421–434. 21263457 10.1038/cr.2011.14 PMC3193428

[R91] Tanaka T, Knapp D, and Nasmyth K (1997). Loading of an Mcm protein onto DNA replication origins is regulated by Cdc6p and CDKs. Cell 90, 649–660. 9288745 10.1016/s0092-8674(00)80526-7

[R92] Tazuke SI, Schulz C, Gilboa L, Fogarty M, Mahowald AP, Guichet A, Ephrussi A, Wood CG, Lehmann R, and Fuller MT (2002). A germline-specific Gap junction protein required for survival of differentiating early germ cells. Development 129, 2529–2539. 11973283 10.1242/dev.129.10.2529

[R93] Tran V, Lim C, Xie J, and Chen X (2012). Asymmetric division of *Drosophila* male germline stem cell shows asymmetric histone distribution. Science 338, 679–682. 23118191 10.1126/science.1226028 PMC3532436

[R94] Tulina N, and Matunis E (2001). Control of stem cell self-renewal in *Drosophila* spermatogenesis by JAK-STAT signaling. Science 294, 2546–2549. 11752575 10.1126/science.1066700

[R95] Valouev A, Johnson SM, Boyd SD, Smith CL, Fire AZ, and Sidow A (2011). Determinants of nucleosome organization in primary human cells. Nature 474, 516–520. 21602827 10.1038/nature10002 PMC3212987

[R96] Van Doren M, Williamson AL, and Lehmann R (1998). Regulation of zygotic gene expression in *Drosophila* primordial germ cells. Curr. Biol 8, 243–246. 9501989 10.1016/s0960-9822(98)70091-0

[R97] Venkei ZG, and Yamashita YM (2015). The centrosome orientation checkpoint is germline stem cell specific and operates prior to the spindle assembly checkpoint in *Drosophila* testis. Development 142, 62–69. 25480919 10.1242/dev.117044

[R98] Venkei ZG, and Yamashita YM (2018). Emerging mechanisms of asymmetric stem cell division. J. Cell Biol 217, 3785–3795. 30232100 10.1083/jcb.201807037 PMC6219723

[R99] Vidaurre V, and Chen X (2021). Epigenetic regulation of *Drosophila* germline stem cell maintenance and differentiation. Dev. Biol 473, 105–118. 33610541 10.1016/j.ydbio.2021.02.003 PMC7992187

[R100] Wang F, and Higgins JM (2013). Histone modifications and mitosis: countermarks, landmarks, and bookmarks. Trends Cell Biol. 23, 175–184. 23246430 10.1016/j.tcb.2012.11.005

[R101] Wei Y, Mizzen CA, Cook RG, Gorovsky MA, and Allis CD (1998). Phosphorylation of histone H3 at serine 10 is correlated with chromosome condensation during mitosis and meiosis in Tetrahymena. Proc. Natl. Acad. Sci. USA 95, 7480–7484. 9636175 10.1073/pnas.95.13.7480 PMC22657

[R102] Wei Y, Yu L, Bowen J, Gorovsky MA, and Allis CD (1999). Phosphorylation of histone H3 is required for proper chromosome condensation and segregation. Cell 97, 99–109. 10199406 10.1016/s0092-8674(00)80718-7

[R103] West JA, Cook A, Alver BH, Stadtfeld M, Deaton AM, Hochedlinger K, Park PJ, Tolstorukov MY, and Kingston RE (2014). Nucleosomal occupancy changes locally over key regulatory regions during cell differentiation and reprogramming. Nat. Commun 5, 4719. 25158628 10.1038/ncomms5719 PMC4217530

[R104] White-Cooper H, Leroy D, Macqueen A, and Fuller MT (2000). Transcription of meiotic cell cycle and terminal differentiation genes depends on a conserved chromatin associated protein, whose nuclear localisation is regulated. Development 127, 5463–5473. 11076766 10.1242/dev.127.24.5463

[R105] Wooten M, Li Y, Snedeker J, Nizami ZF, Gall JG, and Chen X (2020a). Superresolution imaging of chromatin fibers to visualize epigenetic information on replicative DNA. Nat. Protoc 15, 1188–1208. 32051613 10.1038/s41596-019-0283-y PMC7255620

[R106] Wooten M, Ranjan R, and Chen X (2020b). Asymmetric histone inheritance in asymmetrically dividing stem cells. Trends Genet. 36, 30–43. 31753528 10.1016/j.tig.2019.10.004 PMC6925335

[R107] Wooten M, Snedeker J, Nizami ZF, Yang X, Ranjan R, Urban E, Kim JM, Gall J, Xiao J, and Chen X (2019). Asymmetric histone inheritance via strand-specific incorporation and biased replication fork movement. Nat. Struct. Mol. Biol 26, 732–743. 31358945 10.1038/s41594-019-0269-z PMC6684448

[R108] Xie J, Wooten M, Tran V, Chen BC, Pozmanter C, Simbolon C, Betzig E, and Chen X (2015). Histone H3 threonine phosphorylation regulates asymmetric histone inheritance in the *Drosophila* male germline. Cell 163, 920–933. 26522592 10.1016/j.cell.2015.10.002 PMC4636931

[R109] Yabuki N, Terashima H, and Kitada K (2002). Mapping of early firing origins on a replication profile of budding yeast. Genes Cells 7, 781–789. 12167157 10.1046/j.1365-2443.2002.00559.x

[R110] Yadlapalli S, Cheng J, and Yamashita YM (2011). *Drosophila* male germline stem cells do not asymmetrically segregate chromosome strands. J. Cell Sci 124, 933–939. 21325028 10.1242/jcs.079798 PMC3048890

[R111] Yadlapalli S, and Yamashita YM (2013). Chromosome-specific nonrandom sister chromatid segregation during stem-cell division. Nature 498, 251–254. 23644460 10.1038/nature12106 PMC3711665

[R112] Yamashita YM (2018). Subcellular specialization and organelle behavior in germ cells. Genetics 208, 19–51. 29301947 10.1534/genetics.117.300184 PMC5753857

[R113] Yamashita YM, Jones DL, and Fuller MT (2003). Orientation of asymmetric stem cell division by the APC tumor suppressor and centrosome. Science 301, 1547–1550. 12970569 10.1126/science.1087795

[R114] Yamashita YM, Mahowald AP, Perlin JR, and Fuller MT (2007). Asymmetric inheritance of mother versus daughter centrosome in stem cell division. Science 315, 518–521. 17255513 10.1126/science.1134910 PMC2563045

[R115] Yim H, and Erikson RL (2010). Cell division cycle 6, a mitotic substrate of polo-like kinase 1, regulates chromosomal segregation mediated by cyclin-dependent kinase 1 and separase. Proc. Natl. Acad. Sci. USA 107, 19742–19747. 21041660 10.1073/pnas.1013557107 PMC2993418

[R116] Yu C, Gan H, Han J, Zhou ZX, Jia S, Chabes A, Farrugia G, Ordog T, and Zhang Z (2014). Strand-specific analysis shows protein binding at replication forks and PCNA unloading from lagging strands when forks stall. Mol. Cell 56, 551–563. 25449133 10.1016/j.molcel.2014.09.017 PMC4362665

[R117] Yuan Z, Schneider S, Dodd T, Riera A, Bai L, Yan C, Magdalou I, Ivanov I, Stillman B, Li H, and Speck C (2020). Structural mechanism of helicase loading onto replication origin DNA by ORC-Cdc6. Proc. Natl. Acad. Sci. USA 117, 17747–17756. 32669428 10.1073/pnas.2006231117 PMC7395460

[R118] Zaveri L, and Dhawan J (2018). CyclinG to meet fate: connecting pluripotency to the cell cycle. Front. Cell Dev. Biol 6, 57. 29974052 10.3389/fcell.2018.00057 PMC6020794

[R119] Zhang Y, Sun Z, Jia J, Du T, Zhang N, Tang Y, Fang Y, and Fang D (2021). Overview of histone modification. Adv. Exp. Med. Biol 1283, 1–16. 33155134 10.1007/978-981-15-8104-5_1

[R120] Zion EH, Chandrasekhara C, and Chen X (2020). Asymmetric inheritance of epigenetic states in asymmetrically dividing stem cells. Curr. Opin. Cell Biol 67, 27–36. 32871437 10.1016/j.ceb.2020.08.003 PMC7736099

