# Supplementary material for: Differential condensation of sister chromatids acts with Cdc6 to ensure asynchronous S-phase entry in Drosophila male germline stem cell lineage
Source: Dev Cell. Author manuscript; Available in PMC 2023 May 9. (PMC9134767; doi:10.1016/j.devcel.2022.04.007)
Supplement: Supp Info [file NIHMS1798015-supplement-Supp_Info.pdf]

Developmental Cell, Volume *57*

Supplemental information

Differential condensation of sister chromatids acts with Cdc6 to ensure asynchronous S-phase entry in *Drosophila* male germline stem cell lineage

Rajesh Ranjan, Jonathan Snedeker, Matthew Wooten, Carolina Chu, Sabrina Bracero, Taylar Mouton, and Xin Chen

Supplementary figures and figure legends:

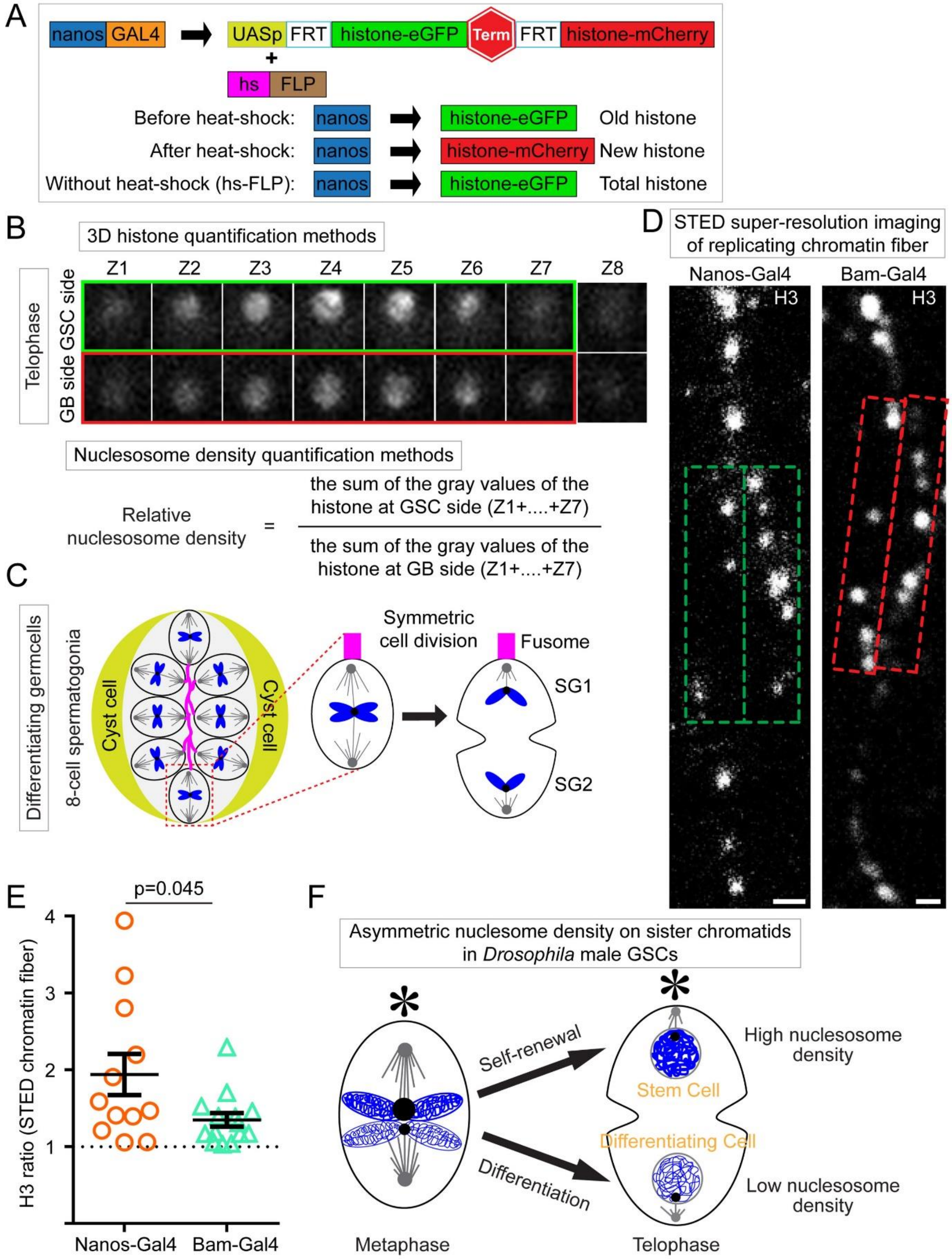

Figure S1. Related to Figure 1: Illustration of the experimental design and quantification method. (**A**) Cartoon depiction of the tag switching approach for differential labeling of old and new histones, and total histone, as well as the *UASp-FRT-histone-eGFP-PolyA-FRT-histone-mCherry-PolyA* transgene, similar to (Tran et al., 2012). UAS, upstream activating sequence; FRT, FLP (flippase) recombination target; histone, H3, H4 and H2A driven by *nanos-Gal4*, an early-stage germline specific driver; *hs-FLP*, the yeast FLP recombinase controlled by the *heat shock* (*hs*) promoter. (**B**) Description of the sum of slices quantification method for calculating relative nucleosome density in a telophase GSC. (**C**) Description of the method used to define the two SGs for quantification purposes based on proximity to the fusome. Even though SGs are thought to be equivalent, they still have a cellular polarity regarding the location of an intercellular structure called fusome. For example, it has been shown that fusome serves as an important organelle to connect SGs and coordinate their cellular responses to DNA damage (Lu and Yamashita, 2017). In the SG cyst, fusomes orient the mitotic spindle so the SG division always results in a daughter cell displaced away from the center group of cells [(Venkei and Yamashita, 2015) and reviewed in (Yamashita, 2018), illustrated here]. Therefore, we defined fusome-proximal SG to be SG1 and the fusome-distal SG to be SG2. And in each of the 8-cell spermatogonial cyst, we averaged all eight ratios of SG1/SG2 for ONE data point in the plot of Figure 1C. (**D**) STED images of early germline-derived chromatin fiber labeled with H3 driven by the *nanos-Gal4* driver (*nanos>H3-GFP*) show the distribution of total H3 during DNA replication. The H3 channel was imaged by STED for EdU-positive fibers. (**E**) Quantification of total H3 distribution from both *nanos-Gal4* labeled early germline-derived and *bam-Gal4* labeled late germline-derived chromatin fibers (Table S5, see Mendeley Data) (*P*= 0.045 by Mann-

Whitney t-test). **(F)** Model describing the nucleosome density asymmetry between sister chromatids during ACD of a GSC.

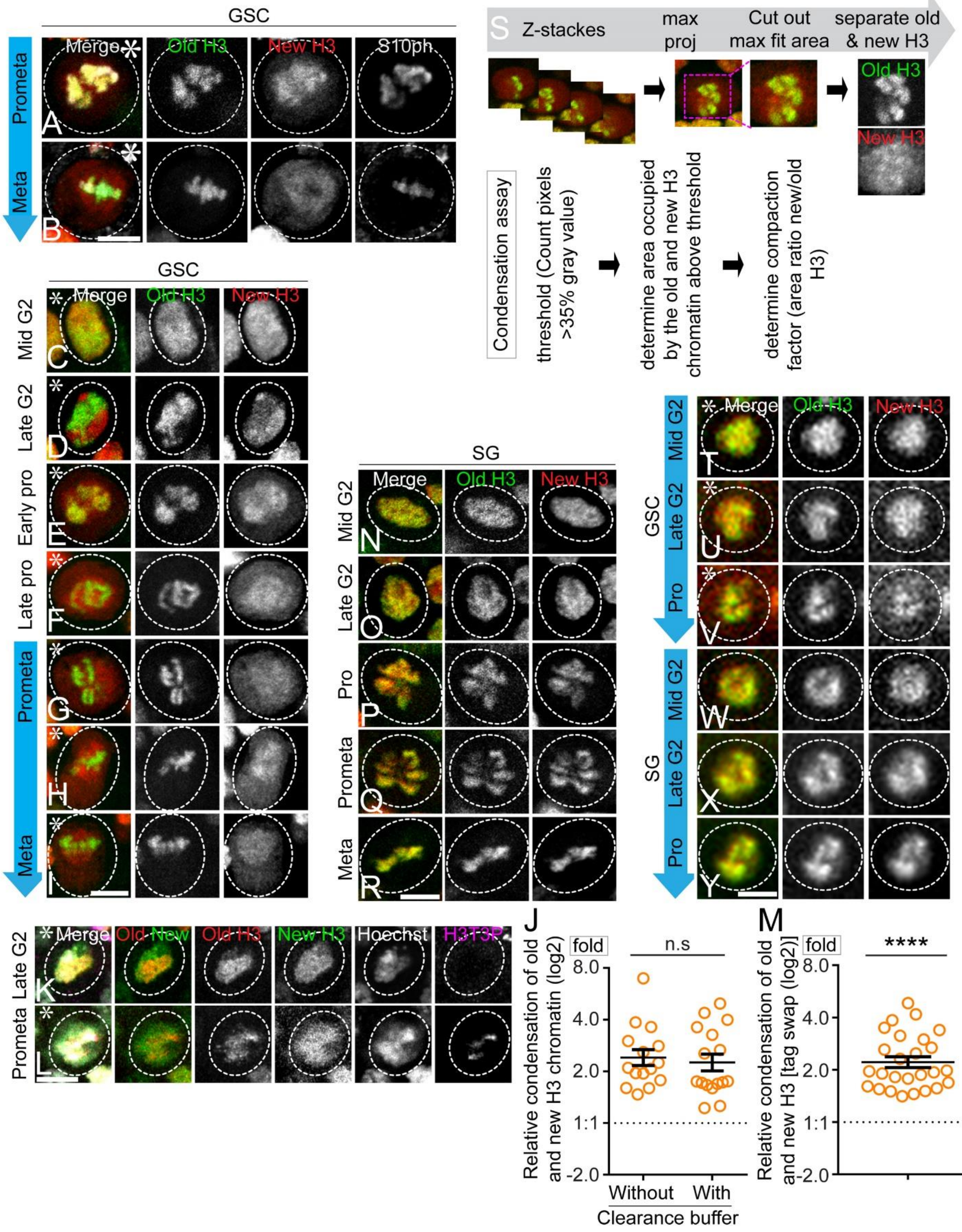
GSC
Merge
Old H3
New H3
S10ph
Prometa
Meta
S
Z-stackes
max proj
Cut out max fit area
separate old & new H3
Condensation assay
threshold (Count pixels >35% gray value)
determine area occupied by the old and new H3 chromatin above threshold
determine compaction factor (area ratio new/old H3)
Mid G2
Late G2
Early pro
Late pro
SG
Pro
Old New
Hoechst
H3T3P
J
fold
n.s
Relative condensation of old and new H3 chromatin (log2)
Without
With
Clearance buffer
M
****
Relative condensation of old and new H3 [tag swap (log2)]
8.0
4.0
2.0
1:1
-2.0

Figure S2. Related to Figure 2: Differential condensation of old versus new H3-enriched regions in GSCs and illustration of the condensation assay. (**A**-**B**) Fixed cell images of old (eGFP, green) and new (mCherry, red) H3 in GSCs at prometa-to-metaphase. (**C-I**) A detailed high-resolution progression of differential condensation throughout mitosis showing that old histone enriched chromatids condense first and remained more condensed through metaphase in GSCs. (**J**) Relative condensation folds between old and new histone enriched regions with and without clearance buffer, showing no significant difference by Mann-Whitney t-test. **(K-L**) Fixed images of old (mCherry, red) and new (eGFP, green) H3 in GSCs at late G2 phase (**K**) and prometaphase (**L**). (**M**) Quantification of the relative condensation difference between old (mCherry, red) and new (eGFP, green) H3-enriched chromatin in GSCs, using fixed cell imaging [GSC: $\log_2$=1.147 ± 0.103], *****P* <10-4 by One-sample t-test. (**N-R**) High spatial resolution imaging showed that old and new histone have comparable condensation from G2 to M phase in symmetrically dividing SGs. (**S**) Illustration of the method used for quantifying the compaction factor between old and new H3 enriched regions by thresholding. (**T-Y**) Live cell imaging showed distinguishable separation between old and new histones from late G2 to prophase GSCs (**T-V**), but not in SGs at the comparable stages (**W-Y**). Scale bars: 2μm. Asterisk: hub.

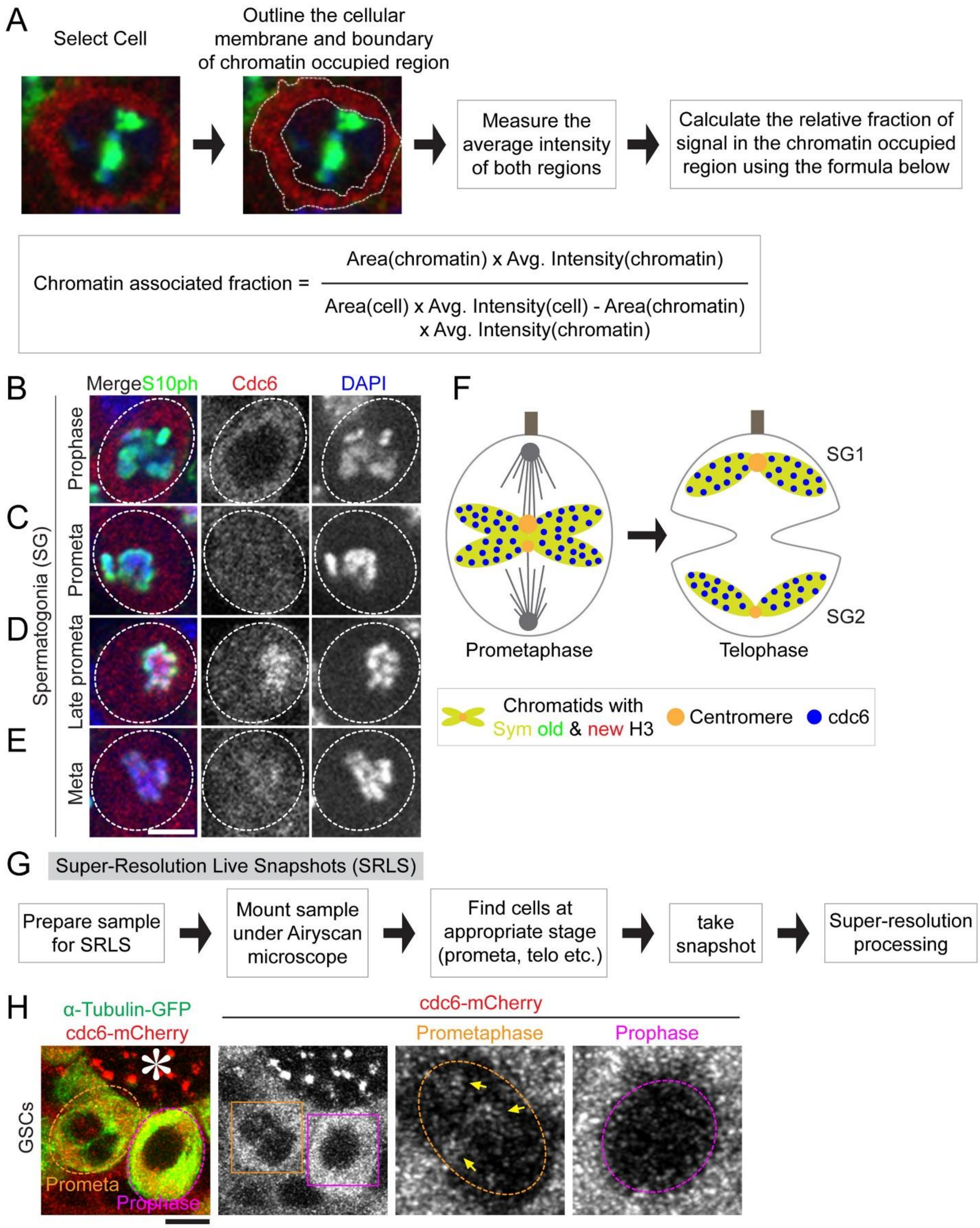

Figure S3. Related to Figure 3: Imaging and quantification method of Cdc6 association with chromatin and segregation patterns. (**A**) Description of the method used to quantify the

relative chromatin associated Cdc6 during mitosis. (**B**-**E**) In SGs, Cdc6 is initially localized in the cytoplasm during prophase (**B**), and starts to associate with the mitotic chromosomes during prometaphase (**C**, **D**) and metaphase (**E**). (**F**) A model for the symmetric binding and segregation of Cdc6 with sister chromatids in SGs. (**G**) Description of the protocol used for Super-Resolution Live Snapshots (SRLS), details in (Ranjan and Chen, 2021). (**H**) Image of prophase and prometaphase GSCs using SRLS. Scale bars: 5μm. Asterisk: hub.

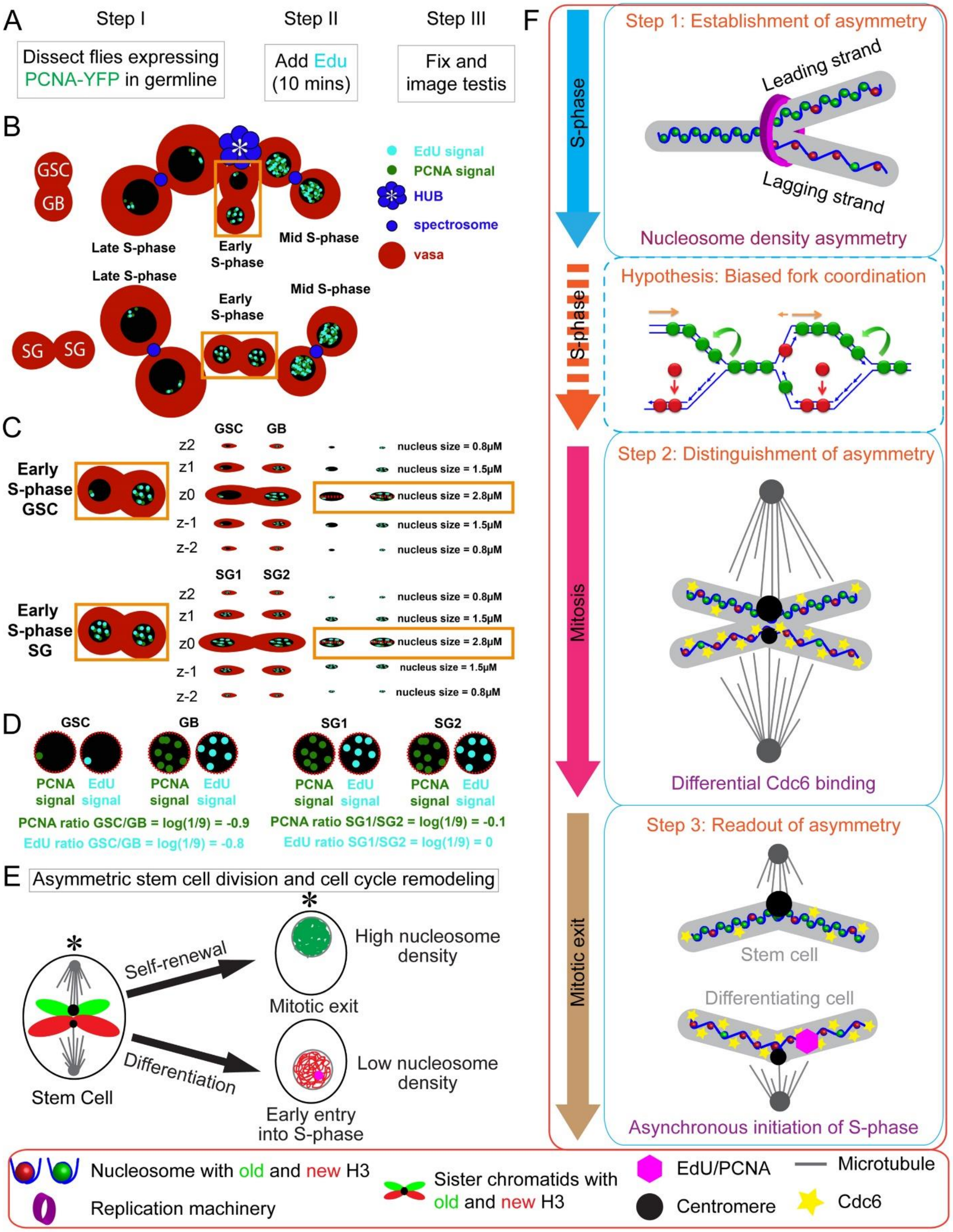

A
Step I
Dissect flies expressing PCNA-YFP in germline
Step II
Add Edu (10 mins)
Step III
Fix and image testis
B
GSC
GB
Late S-phase
Early S-phase
Mid S-phase
EdU signal
PCNA signal
HUB
spectrosome
vasa
SG
C
Early S-phase GSC
Early S-phase SG
GSC GB
SG1 SG2
z2 z1 z0 z-1 z-2
nucleus size = 0.8μM
nucleus size = 1.5μM
nucleus size = 2.8μM
D
PCNA signal
EdU signal
PCNA ratio GSC/GB = log(1/9) = -0.9
EdU ratio GSC/GB = log(1/9) = -0.8
PCNA ratio SG1/SG2 = log(1/9) = -0.1
EdU ratio SG1/SG2 = log(1/9) = 0
E
Asymmetric stem cell division and cell cycle remodeling
Self-renewal
Differentiation
Stem Cell
High nucleosome density
Mitotic exit
Low nucleosome density
Early entry into S-phase
F
S-phase
Mitosis
Mitotic exit
Step 1: Establishment of asymmetry
Leading strand
Lagging strand
Nucleosome density asymmetry
Hypothesis: Biased fork coordination
Step 2: Distinguishment of asymmetry
Differential Cdc6 binding
Step 3: Readout of asymmetry
Stem cell
Differentiating cell
Asynchronous initiation of S-phase
Nucleosome with old and new H3
Replication machinery
Sister chromatids with old and new H3
EdU/PCNA
Centromere
Microtubule
Cdc6

Figure S4. Related to Figure 4: Assessment of S-phase entry in GSC-GB pairs and SG-SG pairs. (**A**) Experimental design: Dissect flies expressing PCNA-YFP in the germline and add 10µM EdU for 10 minutes, followed by fixation and immunostaining. (**B**) S-phase cells can be identified in the testis by the presence of EdU and PCNA foci. Germ cells can be further identified by Vasa signals. GSC-GB pairs were identified by shared cytoplasm and/or the spectrosome structure that connects them. Early S-phase cells show smaller cell bodies and nuclei. GSCs are identified by Vasa-expressing germ cells with direct attachment to the hub cells (HUB, asterisk). Early S-phase GSC-GB show asymmetric PCNA and EdU in the GB nucleus, whereas mid and late S-phase GSC-GB show roughly symmetric distribution of both PCNA and EdU. SG-SG pairs show symmetric distribution of EdU and PCNA during early, mid and late S-phase. (**C**) To quantify signal levels between cells or nuclei, z-stacks were taken of replicating *Drosophila* germ cells, including GSC-GB pairs and SG-SG pairs. The z-slice with the largest nuclear diameter (z0), was taken and used for subsequent steps of quantification. Nuclear size was assessed by measuring diameter (µm) from one side of the nucleus to the opposite side using the imaging processing program FIJI (dashed red line). (**D**) To assess PCNA and EdU signal, channels were separated, and a region of interest was drawn around the nucleus (dashed red circle) using the imaging processing program FIJI. PCNA and EdU signal was quantified as arbitrary fluorescence units. The log value of the fluorescence signal ratio of (GSC/GB) or (SG1/SG2) was taken to give a numerical assessment of the distribution patterns. (**E**) A cartoon model of the GSC-GB pair nuclei to asynchronously enter the next S-phase after asymmetric GSC division. (**F**) A model for how asymmetric histone establishment (Step 1) and segregation (Step 2) could act upstream of the asynchronous entry into the next cell cycle *via* differential

binding of Cdc6 (Step 3). A biased fork coordination step is hypothesized to occur in between Step 1 and 2, which is under current investigation and no data is shown in this work.

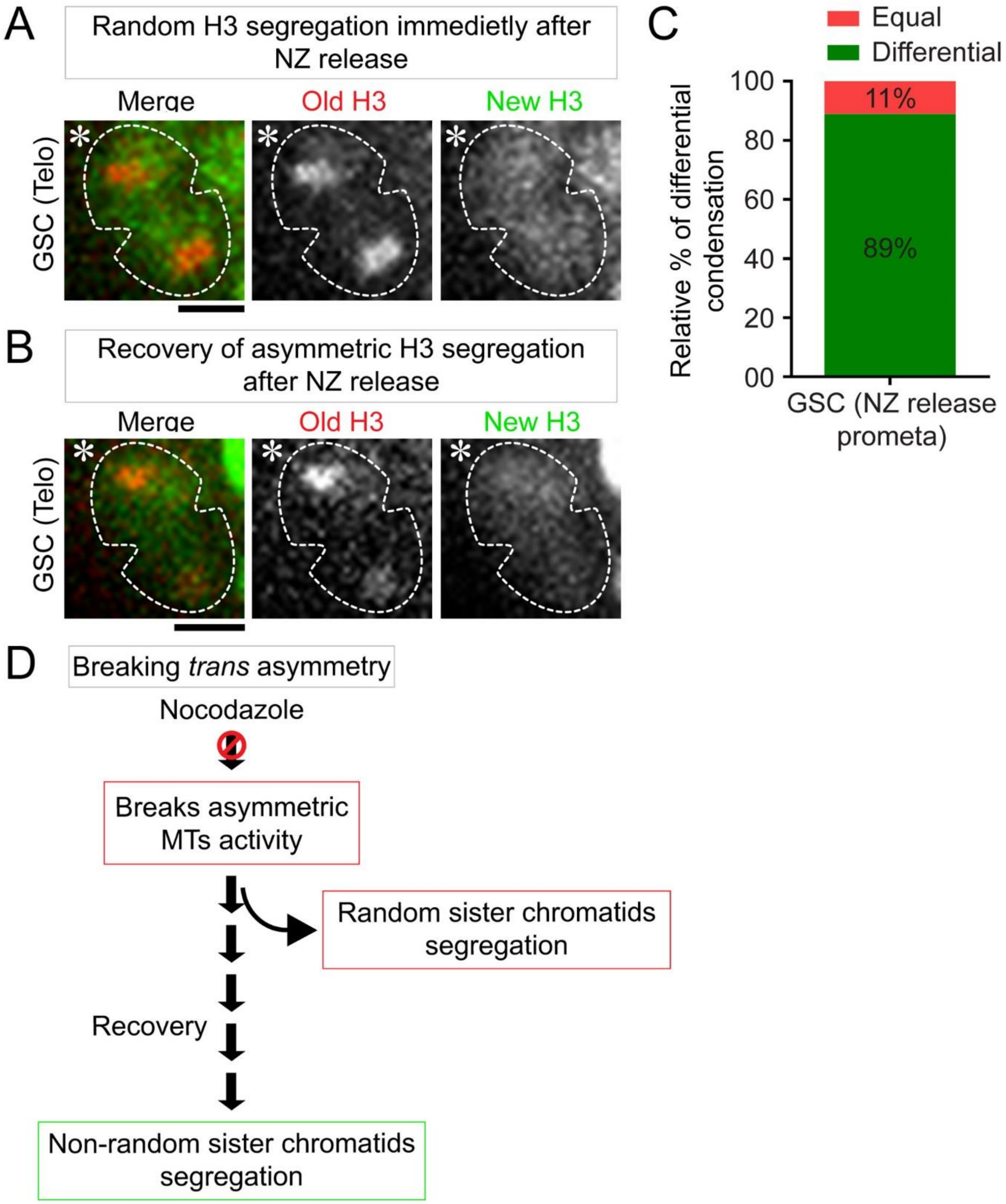

Figure S5. Related to Figure 5: Analyses of the NZ-induced effects on old versus new histone H3 segregation at anaphase or telophase and differential condensation at prometaphase. (**A**) Live cell image of symmetric H3 distribution immediately following release from NZ. (**B**) Live cell image after recovery from NZ showing asymmetric old histone. (**C**) Quantification of the percantage of GSCs with equal versus differential condensation of old versus new histone H3 at prometaphase GSCs, the cutoff for calling equal versus differential condensation was described in the Methods. (**D**) A model of randomization immediately after washing out NZ and recovery after washing out NZ. Scale bars: 5μm. Asterisk: hub.

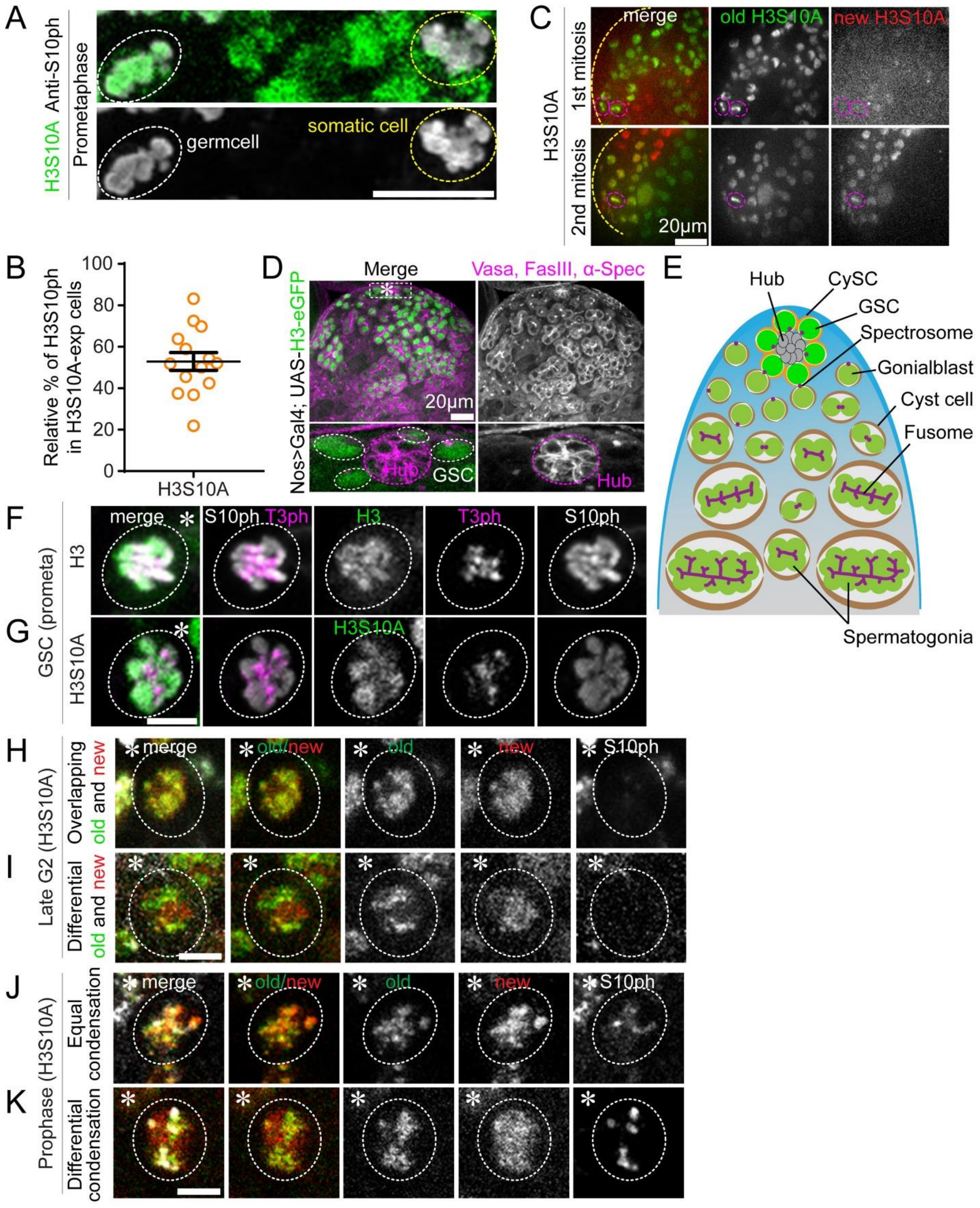

Figure S6. Related to Figure 6: Analyses of H3S10A expression on H3S10 phosphorylation and differential condensation between old and new histones. (**A**) Image of an adjacent mitotic

somatic (cyst stem cell, CySC) and germ cell in the *nos> H3S10A-GFP* testis showing that the level of H3S10ph (gray) is reduced in the early-stage germ cells expressing H3S10A (green). In these experiments, the *H3S10A-GFP* transgene is only expressed in the germ cells but not in the neighboring somatic cells. As H3S10ph is enriched in mitotic cells including both germ cells and somatic cells, the mitotic germ cell (white outlined) shows both H3S10A (green) and H3S10ph (gray), while the mitotic CySC (yellow outlined) only shows H3S10ph (gray) but not H3S10A (green). On the other hand, germ cells at interphase only show H3S10A (green) but not H3S10ph (gray). (**B**) Quantification of the relative levels of H3S10ph between H3S10A-expressing germ cells and the adjacent wild-type somatic cells shows the reduction of H3S10ph levels in the H3S10A-expressing cells [average = 52.88 ± 4.31 (n=14)]. (**C**) Representative images showing that the new H3S10A (red) incorporation dynamics visualized 10-12 hours (1st mitosis) and 22-24 hours (2nd mitosis) after heat-shock induced tag switch from EGFP-H3S10A (old) to mCherry-H3S10A (new). (**D**) *nos>H3-EGFP*-expressing testes were stained with FasIII (hub marker), Vasa (germ cells marker), and a-Spectrin (spectrosome in GSC and GB/fusome in SGs) to distinguish the hub (asterisk), germline stem cells (GSCs), and differentiating germ cells (SGs). (**E**) A cartoon diagram of the testis tip shows the anatomy of different cell types and staged germ cells. (**F-G**) Images showing H3Tph signals in the wild-type H3-expressing GSC (**F**) and the mutant H3S10A- expressing GSC (**G**) at prometaphase. (**H-I**) H3S10A expression results in heterogeneous separation patterns of old versus new histones in late G2 phase GSCs, showing both overlapping (**H**) and differential (**I**) separation patterns. (**J-K**) H3S10A expression results in heterogeneous condensation patterns between old versus new histones in prophase GSCs, showing both equal (**J**) and differential (**K**) condensation patterns. Scale bars: 10µm (**A**), 2µm (**H-K**). Asterisk: hub. All ratios = average ± SE.

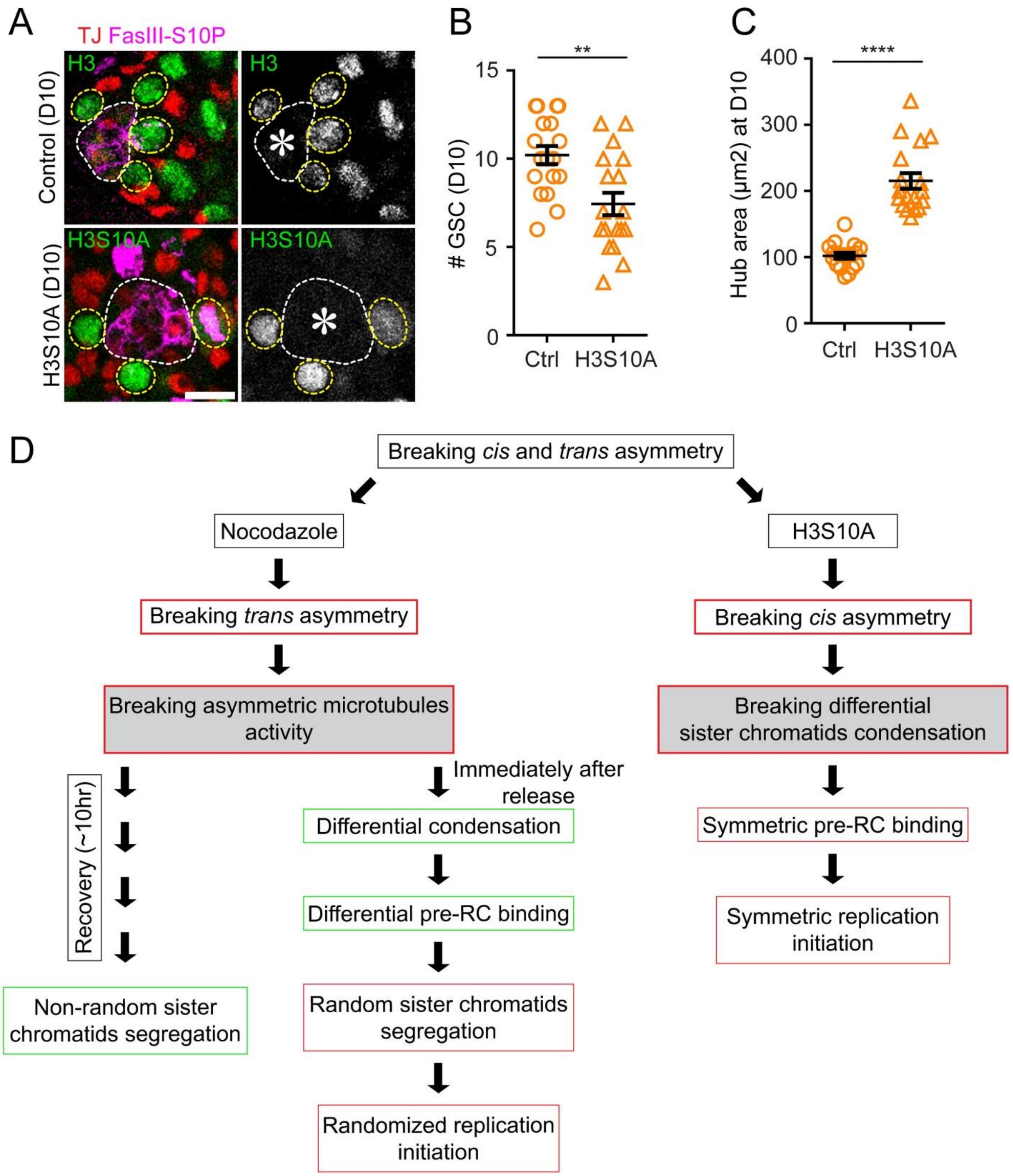

Figure S7. Related to Figure 7: Quantification of GSC and niche defects as well as summary of effects caused by NZ treatment and H3S10A expression. (**A**) Images showing a decreased number of GSCs ten days (D10) after shifting flies from permissive to restrictive

temperature for $Gal80^{ts}$ to turn on *nanos-Gal4* to express H3S10A, compared to D10 after expressing the control H3. (**B**) Quantification showing the reduced number of GSCs at D10 [Ctrl, average = 10.22 ± 0.51 (n= 18); H3S10A, average = 7.44 ± 0.64 (n=18), ***P*< 0.01, Mann Whitney test]. (**C**) Quantification of hub size at D10 showing the expanded hub phenotype [Ctrl, average = 102.29 ± 4.49 (n=18); H3S10A, average = 215.41 ± 11.80 (n=18), *****P*< 0.0001, Mann Whitney test]. (**D**) Comparison of the proposed mechanisms underlying the NZ results in randomizing the entry of the GSC-GB pair nuclei into the next S-phase, while the H3S10A results in inducing symmetric entry of the GSC-GB pair nuclei into the next S-phase. Scale bars: 5 μm. Asterisk: hub. All ratios = average ± SE.
